# Imp/IGF2BP and Syp/SYNCRIP temporal RNA interactomes uncover combinatorial networks of regulators of *Drosophila* brain development

**DOI:** 10.1126/sciadv.adr6682

**Published:** 2025-02-07

**Authors:** Jeffrey Y. Lee, Niles Huang, Tamsin J. Samuels, Ilan Davis

**Affiliations:** ^1^ School of Molecular Biosciences, College of Medical, Veterinary and Life Sciences, University of Glasgow, Glasgow G12 8QQ, UK.; ^2^ Department of Biochemistry, University of Oxford, Oxford OX1 3QU, UK.; ^3^ Lewis-Sigler Institute for Integrative Genomics, Princeton University, Princeton, NJ 08540, USA.; ^4^ Department of Genetics, University of Cambridge, Cambridge CB2 3EH, UK.; ^5^ Department of Physiology, Development and Neuroscience, University of Cambridge, Cambridge CB2 3EL, UK.

## Abstract

Temporal patterning of neural progenitors is an evolutionarily conserved mechanism generating neural diversity. In *Drosophila*, postembryonic neurogenesis requires the RNA binding proteins (RBPs) Imp/IGF2BP and Syp/SYNCRIP. However, how they coachieve their function is not well understood. Here, we elucidate the in vivo temporal RNA interactome landscapes of Imp and Syp during larval brain development. Imp and Syp bind a highly overlapping set of conserved mRNAs encoding proteins involved in neurodevelopment. We identify transcripts differentially occupied by Imp/Syp over time, featuring a network of known and previously unknown candidate temporal regulators that are post-transcriptionally regulated by Imp/Syp. Furthermore, the physical and coevolutionary relationships between Imp and Syp binding sites reveal a combinatorial, rather than competitive, mode of molecular interplay. Our study establishes an in vivo framework for dissecting the temporal coregulation of RBP networks as well as providing a resource for understanding neural fate specification.

## Teaser

RNA binding protein combinations control neuronal identity through a network of temporal regulators.

## INTRODUCTION

Understanding how a small population of neural stem cells (NSCs) generates the complex brain is a key question in developmental biology with substantial biomedical implications, as many neurological disorders stem from aberrant neurodevelopment (*1*, *2*). *Drosophila melanogaster* serves as an excellent model for brain development studies, as it shares many key features with mammalian neurogenesis, including NSC delamination (*3*), the balance between NSC self-renewal and differentiation (*4*, *5*), and tumorigenesis upon uncontrolled proliferation of progenitors (*6*–*8*). The principle of increasing neural diversity through temporally expressed factors is also conserved in *Drosophila*, making it an ideal model to study this mechanism (*9*–*14*). In this process, molecular signatures of upstream NSCs underpin the morphological diversity of immature neurons (*15*). Therefore, elucidating the complex landscapes of gene expression regulation is a critical step in understanding specification of neuronal identity.

Temporal patterning of neuroblasts (NBs; *Drosophila* NSCs) has been most extensively studied in the embryonic ventral nerve cord (VNC) where cascades of temporal transcription factors (TFs) generate birth order–dependent neural diversity (*11*). This involves cross-regulatory control between temporal TFs that drive fate transitions (*9*). Recently, post-transcriptional regulation via RNA stability, translation, and localization has emerged as another layer of diversity generation. RNA binding proteins (RBPs) are the key mediators of post-transcriptional regulation, adding complexity and precision to gene expression output (*1*, *16*). Temporal patterning during larval and pupal stages features opposing gradients of conserved RBPs, IGF2 mRNA binding protein (Imp/IGF2BP) and Syncrip (Syp/SYNCRIP), which negatively regulate each other (*17*, *18*). Unlike short-range transitions driven by embryonic temporal TFs, the gradually changing levels of Imp and Syp in postembryonic NB lineages produce a greater number and diversity of cell types (*19*). Thus, the reciprocity between DNA- and RNA-level regulations is likely to be critical to the fate specification program, accommodating the demands of complex adult functions.

Imp and Syp levels are known to influence specification of the mushroom body (*17*, *20*), motor neurons (*21*), the central complex (*22*), and the visual system (*23*). Beyond fate patterning, they also regulate NB quiescence exit (*24*), NB growth and self-renewal (*25*–*27*), NB decommissioning (*28*, *29*), synaptic transmission (*30*–*34*), and fly behavior (*22*, *35*). Furthermore, the protracted nature of Imp and Syp gradients allows integration of extrinsic signals into the intrinsic patterning program (e.g., steroid hormone, Activin, and Notch signaling) (*14*, *36*, *37*). Although some individual downstream targets of Imp and Syp have been characterized in the studies cited above, the complete RNA interactomes and their temporal variations remain unexplored. Recent single-cell RNA sequencing (scRNA-seq) studies have revealed RNAs displaying cell type– and development-specific expressions in the larval brain (*38*–*41*). Therefore, it is pertinent to identify which of these transcripts are targeted by Imp and Syp, as they may represent potential downstream effectors of fate patterning.

Here, we identify the RNA interactomes of Imp and Syp across early and late stages of the developing larval brain. By adapting iCLIP [individual nucleotide-resolution ultraviolet (UV) cross-linking immunoprecipitation] in larval tissues, we uncover in vivo binding sites of Imp and Syp at an unprecedented resolution. Our dataset reveals highly overlapping Imp and Syp targets enriched for gene expression regulators with diverse neurodevelopmental roles. Leveraging the temporal aspect of our dataset, we identify transcripts that are differentially occupied by Imp and Syp across development, uncovering a complex network of temporal regulators or genes with as-yet-unknown fate patterning functions acting downstream of Imp and Syp. Furthermore, we show key examples of Imp/Syp-mediated post-transcriptional regulation that influence gene expression of early- or late-stage neuronal marker transcripts. Last, we profile physical and evolutionary relationships between Imp and Syp binding sites to provide insights into the molecular interplay underpinning their regulatory cassette. Our comprehensive analysis of the downstream landscapes of Imp and Syp can now serve the community in finely dissecting regulatory mechanisms of temporal cell fate specification, as well as providing a resource for studying regulatory interplay between RBPs.

## RESULTS

### Imp and Syp share many RNA targets across postembryonic brain development

**Fig. 1.**
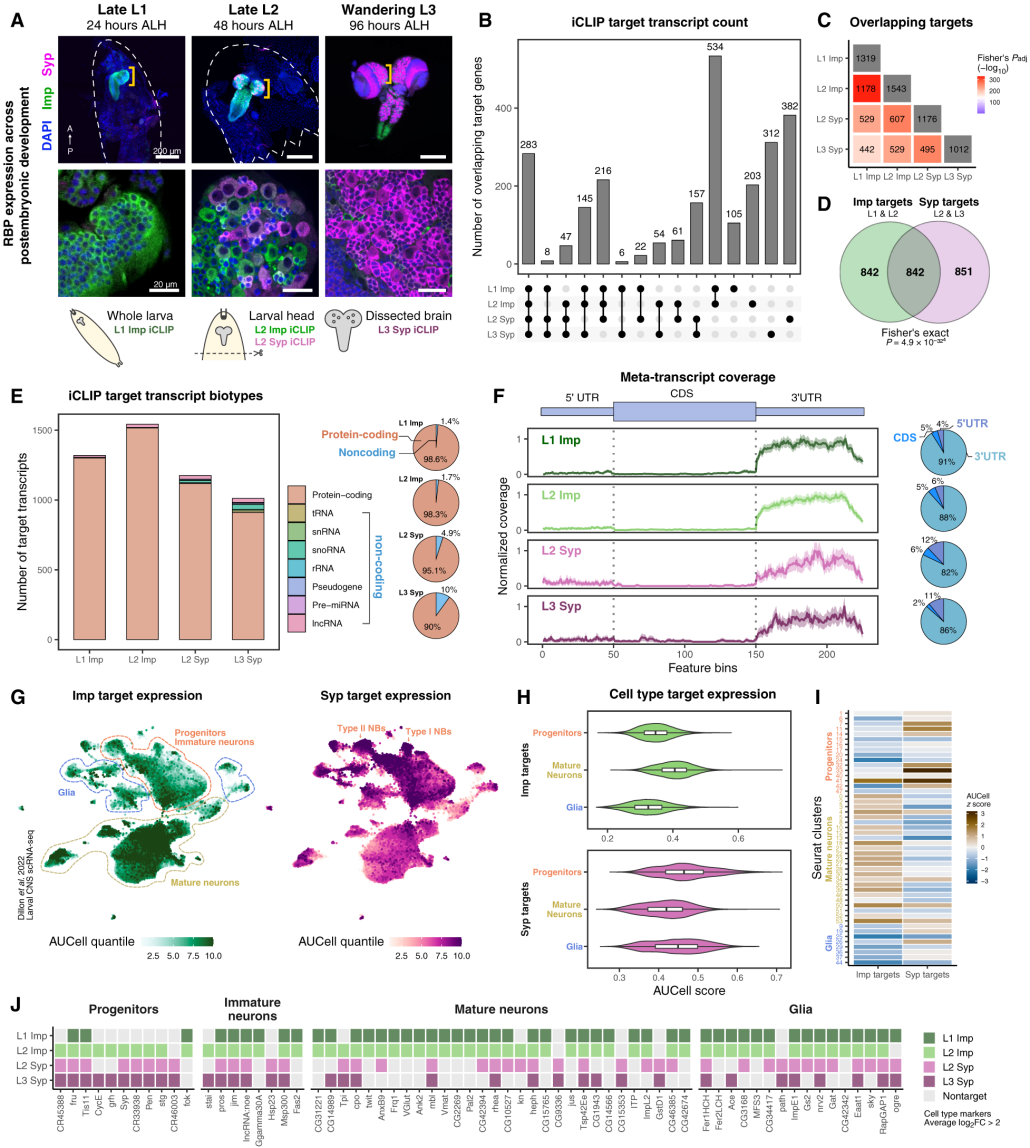
Identification of in vivo RNA targets of Imp and Syp in larval brains. (**A**) Temporal expression pattern of Imp and Syp protein in *Drosophila* larval nervous system. Dotted regions indicate biosamples collected for iCLIP. Anterior (A) and Posterior (P) axis is shown. (**B**) Overlap of Imp and Syp iCLIP target transcripts. (**C**) Statistical significance of target overlap among iCLIP libraries, assessed using the hypergeometric test. (**D**) Statistical significance of target overlap between Imp (L1 and L2 combined) and Syp (L2 and L3 combined) targets. (**E**) Distribution of Imp and Syp iCLIP targets by transcript biotype. The pie chart shows percentages of protein-coding and noncoding target transcripts. (**F**) Meta-transcript coverage of Imp and Syp binding sites on protein-coding genes. The longest CDS and UTR isoform represent each transcript. Pie charts show distributions of RBP binding sites across CDS, 5′UTR, and 3′UTR. (**G**) Area under recovery curve cell (AUCell) scores of Imp and Syp targets across the larval brain scRNA-seq atlas (*39*), indicating relative RBP target expression (see Materials and Methods). Progenitors/immature neurons, mature neurons, and glial cell types identified from the original publication are indicated. (**H**) Comparison of AUCell scores of Imp and Syp targets across progenitors, mature neurons, or glia cell types. (**I**) Heatmap of average AUCell scores of Imp and Syp targets across cell types, with Seurat clusters and their broad cell type (progenitors, immature neurons, mature neurons, and glia) categories indicated. (**J**) Cell type–specific transcripts identified as Imp or Syp targets, including only transcripts identified in at least one iCLIP library. tRNA, transfer RNA; snRNA, small nuclear RNA; snoRNA, small nucleolar RNA; rRNA, ribosomal RNA; miRNA, microRNA; lncRNA, long noncoding RNA; FC, fold change.

To identify in vivo RNA interactomes of Imp and Syp, we adapted iCLIP2 (*42*) for *Drosophila* larval tissues and mapped their transcriptome-wide binding sites. We chose three postembryonic developmental time points: L1, L2, and L3 [24, 48, and 96 hours after larval hatching (ALH)], representing different stages of graded Imp and Syp protein expression levels (Fig. 1A and fig. S1A) (*14*, *17*). For the L1 stage, Imp is highly enriched in the central nervous system (CNS; Fig. 1A); therefore, we immunoprecipitated endogenously tagged Imp::FLAG (*Imp::GFSTF*) from frozen whole-larval powder after UV cross-linking. At the L2 stage, both Imp and Syp proteins are highly enriched in the larval CNS; however, we used severed larval heads to immunoprecipitate both Imp::GFSTF and Syp to avoid contamination from germline-expressed Syp RNP (*43*). For the L3 stage, we individually dissected larval brains and used high-affinity green fluorescent protein (GFP)–trap antibody against endogenously tagged Syp::eGFP (*34*) to maximize RNP recovery. In addition, we prepared corresponding size-matched input libraries (SMInput), which represents the control background (fig. S1B) (*44*). Principal components analysis (PCA) of the sequenced libraries demonstrated clear separation between iCLIP and SMInput libraries and close clustering of biological replicates (fig. S1, C and D), confirming the reproducibility of our experiments. Furthermore, iCLIP reads of Imp and Syp predominantly mapped to 3′ untranslated region (3′UTR) or noncoding RNA (ncRNA) features, while SMInput libraries were overrepresented in coding sequence (CDS), intronic, and intergenic elements, confirming the specificity of the immunoprecipitated RNA fragments (fig. S1E).

We determined nucleotide-resolution binding sites of Imp and Syp by clustering significant cross-links [false discovery rate (FDR) < 0.01] and filtering for enrichment over SMInput samples (*45*, *46*). This analysis identified 1000 to 1500 transcriptome-wide targets of Imp and Syp, harboring significant RBP binding sites (see Materials and Methods, Fig. 1B, and data S1). Of note, the lack of correlation between iCLIP cross-link reads and transcript expression levels suggests that Imp and Syp bind to their targets with specificity rather than passively associating with abundant transcripts (fig. S1F). Notably, we found a significant overlap between Imp and Syp targets (Fig. 1, C and D), indicating a large cohort of transcripts regulated either competitively or cooperatively. Both RBPs primarily targeted protein coding transcripts (Fig. 1E) with a strong preference to 3′UTRs (Fig. 1F), although an increasing proportion of cross-links to ncRNAs was noted in L2 and L3 libraries (Fig. 1E). Since Imp and Syp are highly conserved RBPs across the animal kingdom, we asked whether their RNA targets are also conserved. To this end, we compared our dataset with the RNA targets of human IGF2BP1-3/IMP1-3 in pluripotent stem cells (*47*) and murine Syncrip/hnRNPR in neural cells (*48*, *49*). Within the genes that had high-confidence orthologs (DRSC integrative ortholog prediction tool (DIOPT) score ≥ 8), we found a substantial degree of conservation of RBP targets between fly and mammals: 76% (871 of 1144) for Imp and 55% (623 of 1134) for Syp (data S1). This result suggests evolutionarily conserved RNA binding specificities of Imp and Syp.

Next, we examined the general expression patterns of Imp and Syp targets using recently published larval brain scRNA-seq atlases (*39*, *41*). Grouping cell type clusters into progenitors, immature neurons, and mature neurons, we found a general trend where Imp targets were more highly expressed in mature neurons compared to progenitor cells, while Syp targets were more highly expressed in progenitor cells (Fig. 1, G to I, and fig. S1, G and H). Immature neurons showed intermediate levels of Imp and Syp target expressions. This observation correlates well with spatial RBP expression patterns: Imp is also expressed in early-born functional neurons, while Syp is mainly expressed in NBs and immediate progenies at the L2 stage, where the scRNA-seq was performed (Fig. 1A) (*14*, *39*). Nevertheless, we found that Imp and Syp bind a wide range of cell type marker genes across the neuronal differentiation trajectory as well as glia, indicating their tissue-wide activity (Fig. 1J). Binding sites of Imp and Syp were also found in transcripts specifically expressed in rare NB cell types (e.g., *CycE*, *lncRNA::CR33938*, and *Syp*) (fig. S1, I to J), highlighting the comprehensive cell type representation of our dataset.

### Imp and Syp bind mRNAs encoding regulators of neural development

**Fig. 2.**
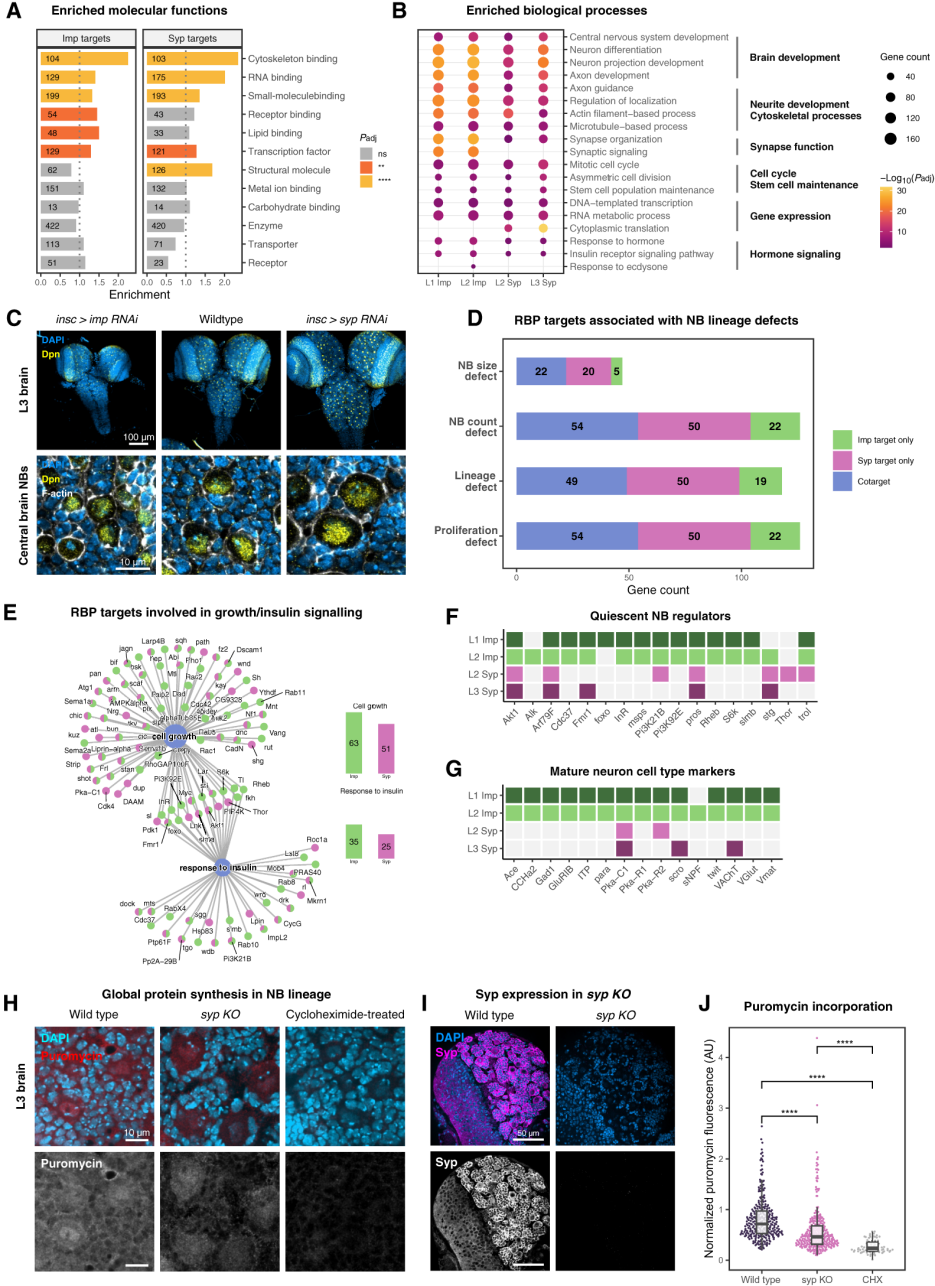
The analysis of Imp and Syp target functions in the NB lineage. (**A**) Enrichments of GO slim molecular function categories for Imp and Syp targets, based on Flybase functional annotations. Background genes were selected from modENCODE larval brain RNA-seq (>10 TPM). Significance was adjusted by the Bonferroni’s method. **P < 0.01 and ****P < 0.0001. (**B**) Dotplot of top enriched GO:Biological Process terms for Imp and Syp iCLIP targets. Semantically similar terms were simplified using the “binary cut” algorithm in the GOSemSim R package. Symbols are not shown for the nonsignificant (ns; adjusted p > 0.05) terms. (**C**) Comparison of L3 brain and NB size in wild-type and *imp* or *syp* knockdown brains using RNAi. The *insc-GAL4* driver targets NBs and immediate progenies. Deadpan (Dpn) marks NBs, and fluorescent phalloidin labels NB cell boundaries. (**D**) Number of Imp and Syp targets involved in regulating NB size, NB number, lineage length, or neural progenitor proliferation, identified via a genome-wide RNAi screen (*50*). (**E**) Genes annotated with cell growth and response to insulin, grouped by Imp/Syp target status. Imp and Syp targets are shown in green and magenta, respectively. Bar plots show the number of targets associated with each term. (**F**) Imp and Syp targets encoding regulators of NB quiescence. (**G**) Imp and Syp targets encoding cell type–specific markers for mature neurons. (**H**) Puromycin incorporation in wild-type (*OrR*), *syp knockout* (*KO*) brains (*syp* null allele/*syp* deficiency), and wild-type brains treated with cycloheximide (CHX) at the L3 stage. Puromycin incorporation was visualized by anti-puromycin immunofluorescence. (**I**) Syp protein depletion in L3 *syp KO* brains compared to wild type. (**J**) Quantification of puromycin incorporation in L3 central brain rype I NBs from (H). (*n* = 3). ****P < 0.0001. AU, arbitrary units.

To obtain functional information about Imp and Syp targets, we performed gene ontology (GO) analysis against the brain transcriptome (full GO enrichment analysis output is available in data S2). Both Imp and Syp bind transcripts encoding proteins with diverse molecular functions, with strong enrichments for TF/RBPs involved in gene expression control at transcriptional and post-transcriptional levels and also cytoskeleton regulators (Fig. 2A). For biological processes, many neurogenesis terms were highly enriched for both Imp and Syp targets, covering broad aspects of neural development from NSC differentiation to axon/synapse maturations (Fig. 2B). Loss of Imp results in smaller NBs and underproliferation of NB lineages, while *syp* knockdown results in the opposite overgrowth and larger brain phenotypes (Fig. 2C) (*25*, *27*, *28*). We hypothesized that Imp and Syp give rise to these phenotypes via their downstream targets. Therefore, we intersected our dataset with a genome-wide RNA interference (RNAi) survey for NB self-renewal and proliferation phenotypes (*50*). We found that 20% of the RNAi screen genes (127 of 620) were Imp or Syp targets involved in either NB size, NB number, lineage length, or proliferation defects (Fig. 2D). No particular phenotype class was enriched against the screen dataset distribution (fig. S2A). Overall, this result illustrates the pervasive role of Imp and Syp across NB lineage maintenance and differentiation.

Although Imp and Syp targets were generally enriched for similar biological processes (Fig. 2B), Imp targets were more strongly enriched for “cell growth” and “response to insulin” terms (Fig. 2E). Cellular growth and insulin signaling are pivotal processes involved in reactivation of quiescent cells (*5*, *51*, *52*), and Imp has been shown to influence the timing of NB reactivation in early larval stages (*24*). As expected, many of the NB quiescence regulators were identified as Imp targets (Fig. 2F), suggesting that Imp may act on NB reactivation through these transcripts. Furthermore, we found an enrichment of mature neuron cell type markers (Fig. 2G) (*39*) and “synaptic signaling” GO terms within Imp targets (Fig. 2B), reflecting the spatial expression pattern of Imp in functional neurons of embryonic origin. On the other hand, we identified a large group of transcripts encoding ribosomal proteins that bind specifically to Syp (fig. S2B), suggesting its role in regulating ribosome processing and translation. To test whether Syp affects global translation levels, we performed an ex vivo puromycin incorporation assay that is sensitive to cycloheximide (CHX) treatment (Fig. 2H). Compared to wild-type L3 brains, *syp KO* (*syp^e00286^/Df-BSC124*) brains showed reduced puromycin incorporation, indicating down-regulated protein synthesis in the absence of Syp (Fig. 2, I and J). Together, our analysis reveals broad functions of Imp and Syp targets in the nervous system, as well as key differences between Imp- and Syp-specific target transcripts.

### Imp and Syp dynamically occupy transcripts encoding temporal factors over time

**Fig. 3.**
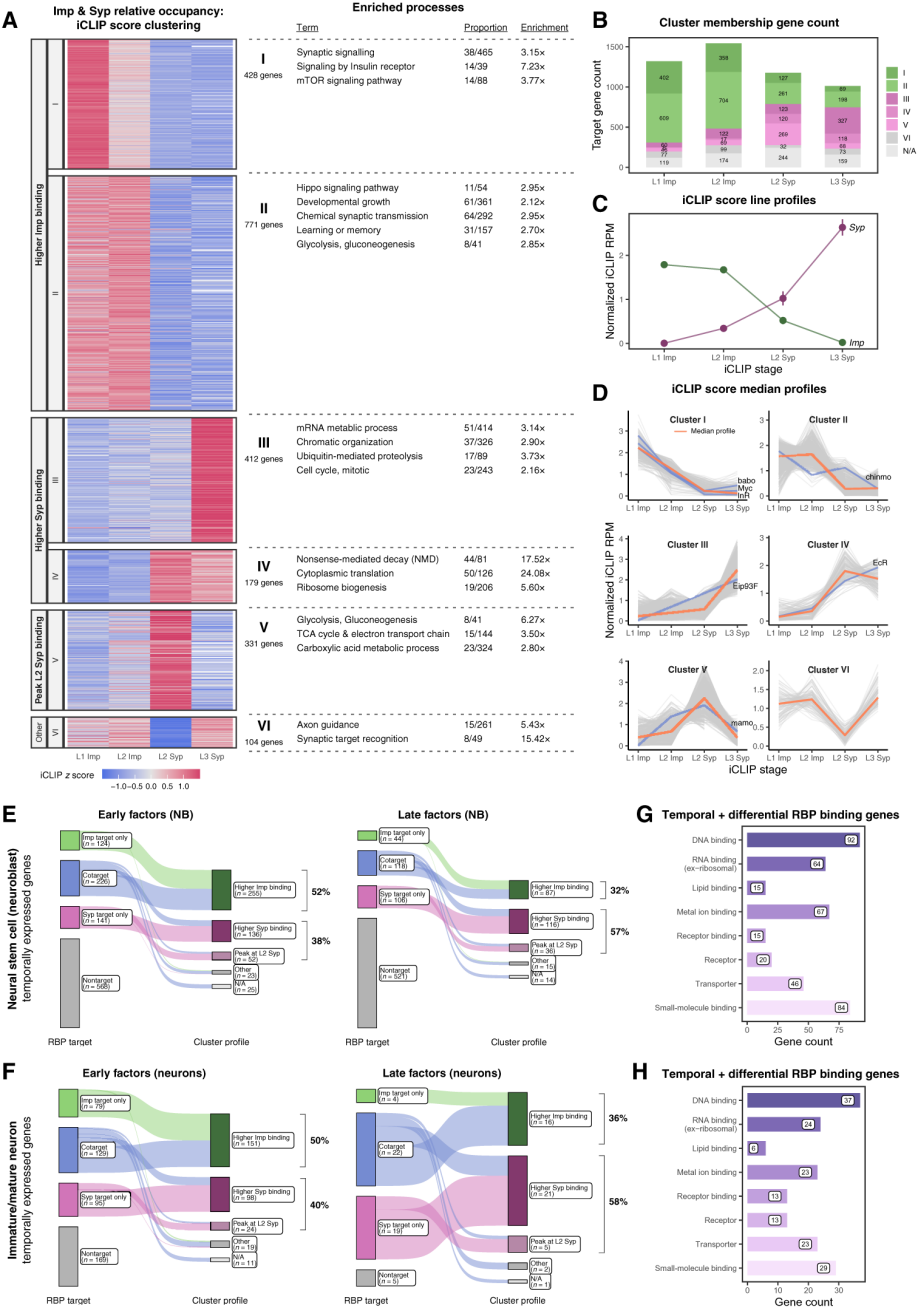
Temporally expressed transcripts interact dynamically with Imp and Syp. (**A**) Heatmap of *k*-means clustering for Imp and Syp relative occupancy (iCLIP score) across developmental stages. Relative occupancy for each gene was calculated by normalizing iCLIP cross-link counts to transcript abundance. Enriched GO, KEGG, and Reactome terms are shown for each cluster. Clusters I and II form the “higher Imp binding” group, while clusters III and IV form the "higher Syp binding" group. (**B**) Distribution of individual iCLIP library targets by cluster. "Higher Imp binding" and "higher Syp binding" groups are shown in shades of green and magenta, respectively. (**C**) Line profiles of Imp and Syp relative occupancy on their own transcripts, showing opposing interaction gradients. (**D**) Grouped line plots of iCLIP score profiles of each cluster. Median profiles are indicated by red lines. Early temporal factors (*babo*, *Myc*, *InR*, and *chinmo*) are in the "higher Imp binding" group, while late temporal factors (*Eip93F* and *EcR*) are in the "higher Syp binding" group. *mamo*, which specifies the middle mushroom body neuronal fate, is in cluster V, showing strong Syp interaction at the L2 stage. (**E**) Sankey plot of intersections between temporally expressed factors in NBs (early and late) and transcripts dynamically occupied by Imp and Syp. Not applicable (N/A) indicates RNA targets that do not show dynamic interaction pattern with Imp and Syp. (**F**) Sankey plot of intersections between temporally expressed factors in immature/mature neurons (early and late) and transcripts dynamically occupied by Imp and Syp. Temporally expressed transcripts are taken from isolated NB RNA-seq or staged larval brain scRNA-seq (*17*, *18*, *39*). (**G** and **H**) Enriched molecular functions of temporally expressed genes dynamically interacting with Imp and Syp in (G) NB and (H) immature/mature neurons. FlyBase GOSlim terms are shown for high-level classification.

The postembryonic neural development program is heavily influenced by the relative levels of Imp and Syp (*9*, *19*). Hence, we hypothesized that RNAs involved in temporal fate patterning and NB behavior may dynamically interact with Imp and Syp over time. To investigate this, we performed a *k*-means clustering based on iCLIP scores of each target that were normalized by their expression levels (full analysis output is available in data S3). Here, we identified six groups of genes displaying distinct binding profiles to Imp and Syp across developmental time points (Fig. 3A). As expected, Imp targets were enriched in clusters I and II (“higher Imp binding”) and Syp targets in clusters III and IV (“higher Syp binding”) (Fig. 3B). However, some Imp targets were also classified as “higher Syp binding” and vice versa, indicating that Imp and Syp can share RNA targets with widely varying occupancy levels (Fig. 3B). Each cluster was associated with distinct biological processes as shown by GO, Reactome, and Kyoto Encyclopedia of Genes and Genomes (KEGG) enrichment analyses (Fig. 3A). For example, growth [Hippo and mammalian target of rapamycin (mTOR) pathways] and signaling-related transcripts had higher Imp occupancy, while RNA metabolizing genes showed stronger interaction with Syp. Of note, we found that energy metabolism terms were overrepresented in "higher Imp binding" and in cluster V genes (Fig. 3A). Cluster V transcripts strongly interact with Syp specifically at the L2 stage (“peak L2 Syp binding”), which represents a key Imp-to-Syp transition period. We hypothesized that genes in the peak L2 Syp binding group might be involved in establishing the early-to-late fate transition. Furthermore, metabolic switch and oxidative phosphorylation have been shown to affect NB temporal patterning and neuronal specifications (*53*, *54*). Consistent with this idea, many glycolytic enzymes and components of the tricarboxylic acid (TCA) cycle dynamically interacted with Imp or specifically with Syp at the L2 stage (fig. S3, A and B), suggesting the role of Imp and Syp in metabolic reprogramming. In contrast, genes in cluster VI, with reduced L2 Syp interactions, were enriched for axonogenesis terms (Fig. 3A), which reflects the lack of Syp expression in this subcompartment at the L2 stage (Fig. 1A).

Next, we investigated whether our dynamic binding analysis could reveal regulators of temporal fates. We found similar opposing gradients of relative RBP occupancy with *imp* and *syp* transcripts reminiscent of their temporal mRNA expressions (Fig. 3C) (*17*). Furthermore, known early temporal factors (e.g., *babo*, *Myc*, and *chinmo*) were identifiable in the "higher Imp binding" group, while late factors (e.g., *Eip93F* and *EcR*) were found in “higher Syp binding" group (Fig. 3D). To expand our exploration, we analyzed dynamic temporal interactors of Imp and Syp that are differentially expressed in early versus late stages of brain development (*17*, *38*, *39*). In NBs, 491 early and 268 late factors were targeted by Imp or Syp (Fig. 3E), and notably, 90% of these transcripts exhibited dynamic RBP binding profiles. We observed a similar trend for temporally expressed genes in neurons (Fig. 3F), and for both cell types, a greater proportion of early factors was classified as “higher Imp binding” while most of the late factors fell under the “higher Syp binding” group. In a specific NB lineage, 8 of 15 de novo TFs influencing Lin A/15 motoneuron diversity were Imp and Syp targets (fig. S3E) (*21*). Notably, their contribution to early-, middle-, and late-born identities aligned with the RBP relative occupancy: Early factors interacted more strongly with Imp and late factors with Syp. Overall, temporal genes were enriched in TFs and RBPs (Fig. 3, G and H, and fig. S3, C and D), suggesting that they may act downstream of Imp and Syp to regulate gene expression and subsequent temporal fates.

NB-derived brain tumors also exhibit a cellular patterning akin to the developmental patterning. For example, *pros*-RNAi–induced tumors progress to generate a hierarchy of fast-dividing Imp^+^/Chinmo^+^ cells and slow-dividing Syp^+^/Eip93F^+^ cells (*55*). Furthermore, both Imp and Syp have been shown to affect proliferation of *brat*-RNAi–mediated tumors (*26*). To contextualize our results, we compared Imp^+^/Chinmo^+^ versus Syp^+^/Eip93F^+^ tumor marker genes on their Imp/Syp relative occupancy group memberships. First, we discovered that a substantial number of tumor cell markers turned out to be Imp and Syp targets (fig. S3, F and G). Here, Imp^+^/Chinmo^+^ marker genes were enriched for the "higher Imp binding" group, while a greater proportion of Syp^+^/Eip93F^+^ markers consisted of "higher Syp binding" group members (fig. S3F). Notably, the majority of the dynamically interacting tumor markers were already identified in the developing NB/neurons (fig. S3G), which suggests that the targets of Imp and Syp could be redeployed when establishing tumor differentiation trajectories.

### Imp and Syp post-transcriptionally regulate temporally expressed transcripts

**Fig. 4.**
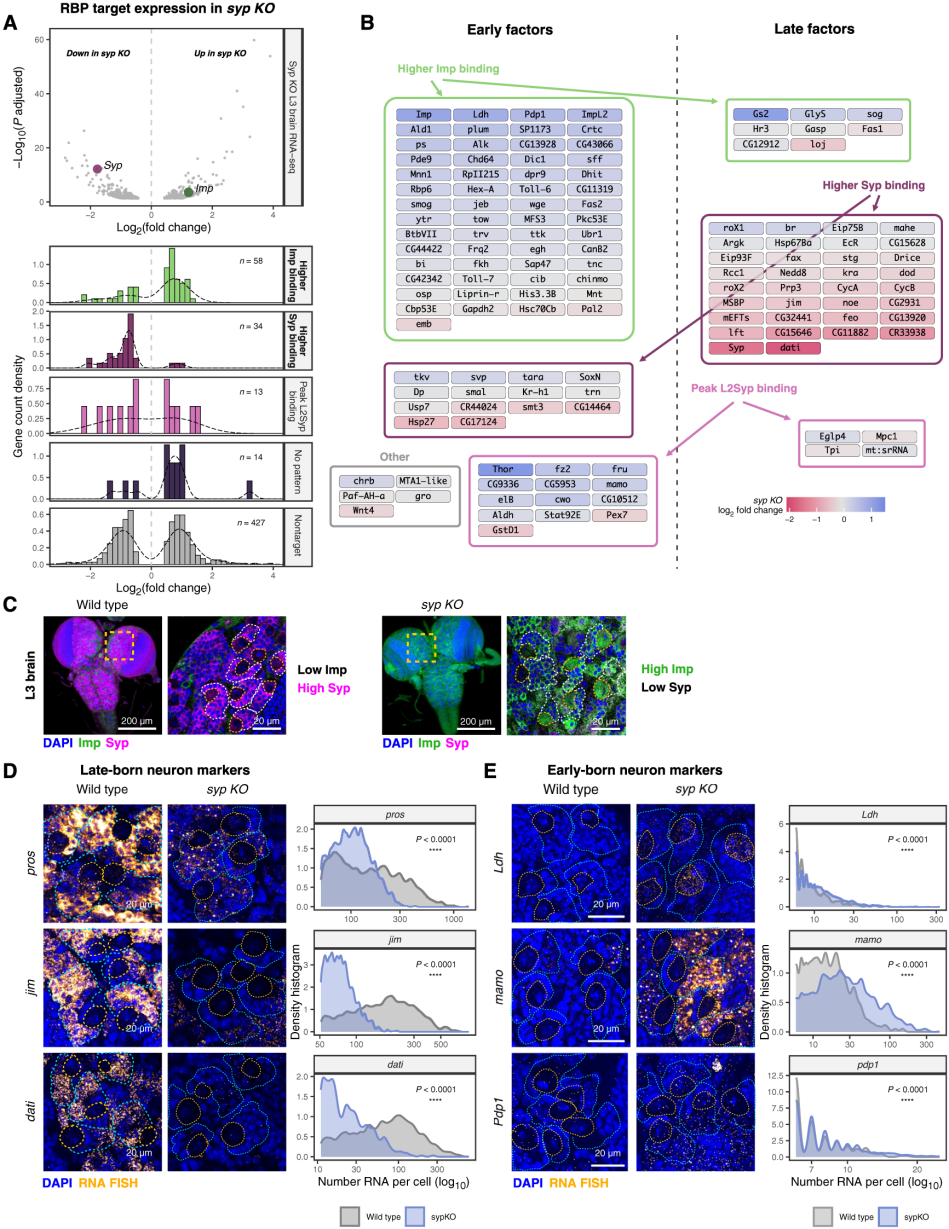
Imp and Syp bind and regulate temporally expressed transcripts. (**A**) RNAs dynamically interacting with Imp and Syp show differential expression in L3 *syp KO* brains. In the *syp KO*, *syp* is down-regulated (magenta symbol), and *imp* is up-regulated (green symbol). Histograms display the log_2_ fold change distribution in transcript abundance for each RBP occupancy group. The *syp KO* brain RNA-seq data were taken from (*35*). (**B**) Network of early and late temporally expressed transcripts dynamically interacting with Imp and Syp. Fill color indicates log_2_ fold change in transcript expression in the *syp KO* brain. Temporal transcripts were taken from staged larval brain scRNA-seq (*38*, *39*). Transcripts with unchanged expression are also shown. (**C**) Expression of Imp and Syp proteins in wild-type and *syp KO* L3 brains, with central brain regions highlighted (blow-up region). Type I NBs and progenies are outlined with dashed lines. (**D**) Altered mRNA expression of late-born (*pros*, *jim*, and *dati*) neuronal markers in wild-type and *syp KO* L3 Type I NB lineages. smFISH images were quantified as the number of mRNAs per cell (log scale), shown as density histogram per genotype. All cells in the field of view were analyzed. NBs and progenies outlined with dashed lines. Kolmogorow-Smirnov test of differential distribution. (*n* = 3). ****P < 0.0001. (**E**) Altered mRNA expression of early-born (*Ldh*, *mamo*, and *Pdp1*) neuronal markers in wild-type and *syp KO* L3 Type I NB lineages. Analysis is the same as late-born markers. (*n* = 3). ****P < 0.0001.

Next, we examined whether Imp and Syp can regulate their downstream targets. We analyzed changes in the target transcript abundance in *syp KO* L3 brain RNA-seq (*35*), a system in which the loss of Syp results in protracted expression of *imp* (fig. S4A). We found that "higher Syp binding" transcripts were generally down-regulated in *syp KO* brains, whereas "higher Imp binding" genes were up-regulated (Fig. 4A). Notably, classification just by Imp or Syp target status showed relatively uniform distributions of up- and down-regulated genes (fig. S4B), indicating that RBP relative occupancy is a better predictor of regulatory trends than the target status alone.

The correlation between transcript abundance and RBP relative occupancy suggests a regulatory network where Imp and Syp control target RNA stability to promote early or late temporal fates (Fig. 4B). Consistently, our analysis recovered mRNAs indicative of early-born differentiating neurons (*mamo*, *Ldh*, and *Pdp1*) and late-born immature neurons (*pros*, *jim*, and *dati*), which were previously identified from staged scRNA-seq studies (Fig. 4B and fig. S4C) (*39*, *56*). However, upstream regulatory roles of Imp and Syp on these transcripts are not fully known. To address this, we used single-molecule fluorescence in situ hybridization (smFISH) in *syp KO* L3 brains to examine the RNA expression of temporal neuronal markers in high Syp versus high Imp conditions (Fig. 4C). In *syp KO* central brain Type I NB lineages, we observed reduced expression of late-born markers (*pros*, *jim*, and *dati*) and up-regulation of early-born transcript markers (*Ldh*, *mamo*, and *Pdp1*) when Imp levels were sustained in the absence of Syp (Fig. 4, D and E). In addition, *insc-GAL4*–driven *imp* knockdown NB lineages yielded a smaller number of immature neurons expressing *Ldh*, *mamo*, and *pdp1* (fig. S4, D and E), which is consistent with the Imp’s role in promoting early-born markers. Notably, many of these changes occurred despite active transcription, as seen in nuclear transcription sites, suggesting altered transcript stability. We also observed that intronic smFISH signals of late-born markers, *pros* and *jim*, appeared as early as 48 hours ALH, but exonic signals accumulated only post–96 hours ALH (fig. S4F), indicating RNA stability control despite an early transcription initiation. This regulatory interaction was also observed in VNC Type I NB lineages (fig. S4, G and H), where Imp and Syp promoted early and late neuronal markers, respectively. A similar trend was also seen in Type II lineages (fig. S4, I and J), with the exception of *jim* and *pdp1*, which showed comparable mRNA levels under modulated Imp/Syp levels. These results suggest that the regulatory role of Imp/Syp on temporally expressed transcripts is shared between multiple neural lineages.

**Fig. 5.**
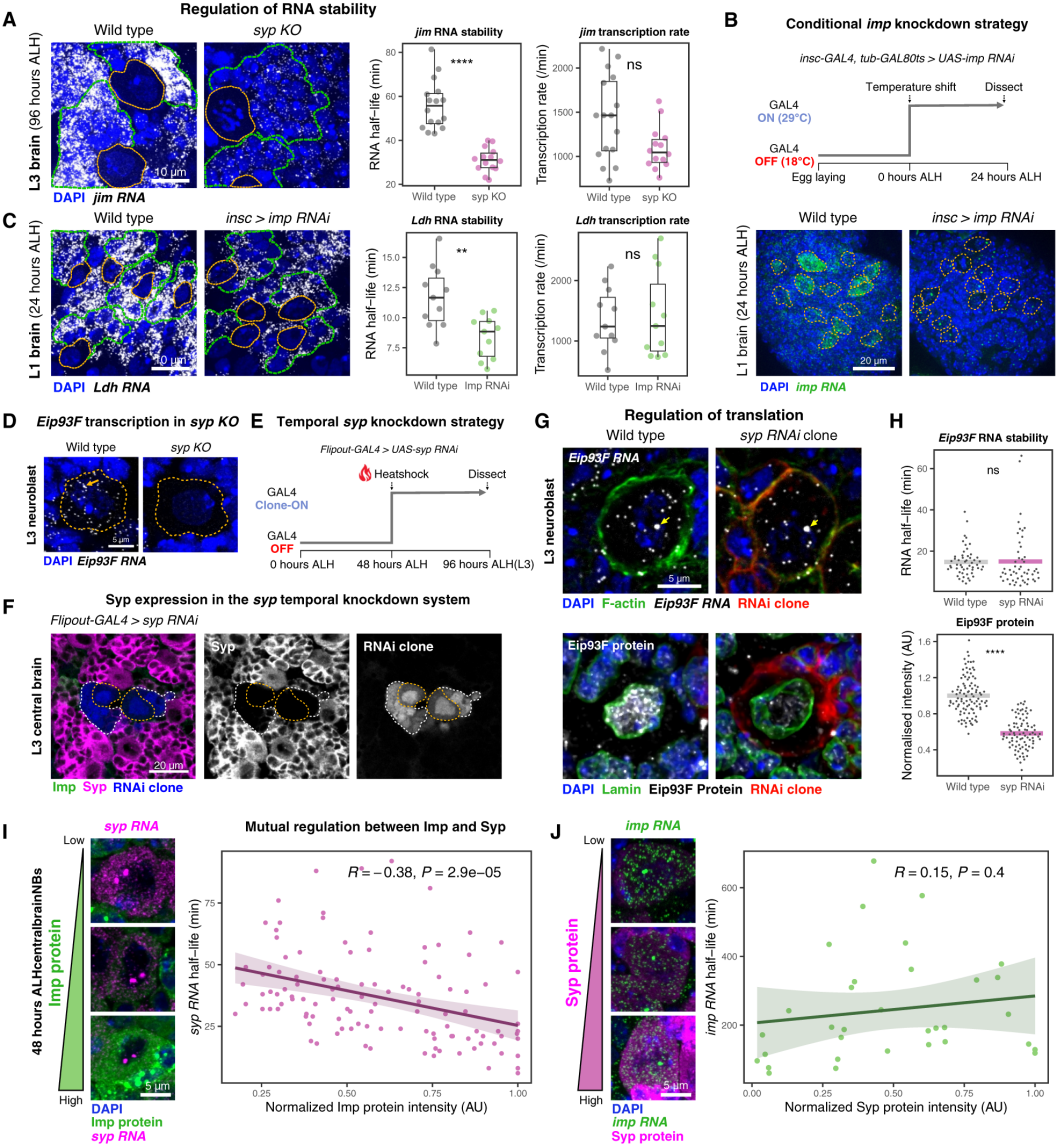
Imp and Syp post-transcriptionally regulate downstream targets. (**A**) Visualization and quantification of *jim* mRNA stability in the absence of *syp*. RNA half-life was calculated by comparing nascent and mature RNA levels in L3 (96 hours ALH) central brain regions (see Materials and Methods). (*n* = 3). ****P < 0.0001. (**B**) Temporal *imp* knockdown strategy using *insc-GAL4* and *tub-GAL80ts*. The smFISH image of L1 *imp* knockdown brain shows active *imp* transcription (bright foci) but reduced mature transcript accumulation. (**C**) Visualization and quantification of *Ldh* mRNA stability in *imp RNAi* temporal knockdown system. RNA half-lives were measured in L1 (24 hours ALH) central brain regions. (*n* = 2). **P < 0.01. (**D**) Loss of *Eip93F* transcription in L3 central brain NBs in *syp KO*. (**E**) Strategy for temporal *syp* knockdown using an inducible *flip-out GAL4* system. (**F**) Validation of *syp* knockdown in temporal *flip-out GAL4 > syp* RNAi NB clones, allowing specific knockdown of Syp without Imp overexpression. (**G**) Assessment of *Eip93F* RNA and protein levels following temporal *syp* removal in central brain Type I NBs. Yellow arrows indicate nuclear transcription sites. Temporal *flip-out GAL4 > syp RNAi* clone system was used for a 48-hour knockdown between 48 and 96 hours ALH. (**H**) Quantification of *Eip93F* RNA half-life and nuclear Eip93F protein levels in Type I NBs after temporal *syp* knockdown. Number of NBs analyzed: RNA stability (wild-type 52 and *syp RNAi* clone 51), protein levels (wild-type 99 and *syp RNAi* clone 87) from three independent biological replicates. ****P < 0.0001. (**I**) Correlation between Imp protein and *syp* RNA half-life in individual NBs at 48 hours ALH. Central brain Type I NBs of Imp::GFSTF genotype with smFISH against *syp*. Pearson correlation test. (*n* = 3). (**J**) Correlation between Syp protein and *imp* RNA half-life in individual NBs at 48 hours ALH. Central brain Type I NBs of Syp::GFP genotype with smFISH against *imp*. Pearson correlation test. (*n* = 2).

We previously demonstrated that Syp can stabilize *pros* mRNA through the extended 3′UTR regulatory sequences (*35*). To characterize a further example, we calculated the RNA half-life of a late-born neuron marker *jim* in *syp KO* L3 brains. We calculated *jim* RNA half-life from smFISH images by relating single-molecule counts of nascent and mature mRNAs (fig. S5, A and B) (*57*, *58*), which yielded a value comparable to an orthogonal metabolic labeling method (*59*). Here, we found a significant destabilization of *jim* in *syp KO* brains (Fig. 5A and fig. S5C), suggesting that Syp is crucial for the correct post-transcriptional regulation of *jim*. Similarly, we tested the role of Imp in regulating an early-born neuron marker *Ldh* in the L1 brain (24 hours ALH). We used a temporal *imp* knockdown system using *insc-*Galactose responsive transcription factor (GAL4) and *tub-*GAL80ts (Fig. 5B) because constitutive knockdown of *imp* results in NB reactivation defects (*24*). In this system, loss of *imp* resulted in the destabilization of *Ldh* in the NB lineage (Fig. 5C and fig. S5C). These results suggest that Imp and Syp can influence expression of early or late neuronal markers through regulating RNA stability of their downstream targets.

Imp and Syp can also affect mRNA translation (*17*, *34*), and this mode of control is not detectable from mutant transcriptomic studies. To characterize an example of regulated translation contributing to temporal patterning, we assessed the gene expression level of *Eip93F*, a temporal TF that interacts genetically with *syp* for late-stage NB specification (*14*, *28*). *Eip93F* mRNA interacts strongly with Syp (fig. S5D); however, its abundance does not significantly change in *syp KO* brains, which suggests invariant RNA stability. Because *Eip93F* transcription in NBs is sensitive to Syp during the Imp-to-Syp transition stage (Fig. 5D and fig. S5E), we assessed mRNA half-life and protein level of Eip93F in a temporal *syp* knockdown system in Type I NBs (Fig. 5, E and F). We found comparable RNA stability of *Eip93F* between wild-type and *syp* RNAi clone NBs, but Eip93F protein was significantly down-regulated in the absence of Syp (Fig. 5, G and H). To assess the regulatory specificity, we measured puromycin incorporation in our temporal *syp* knockdown system, as constitutive *syp KO* affects protein synthesis (Fig. 2J). In *syp* RNAi clones, puromycin fluorescence was only slightly reduced (~7%) compared to the wild-type NBs, suggesting that the global translation level is only mildly affected by the temporal *syp* modulation, unlike a constitutive knockout (fig. S5, F and G). Given the ~50% decrease in Eip93F protein in the temporal system, our results indicate that Syp is specifically required for the correct protein synthesis output of *Eip93F*. Our temporal knockdown system could not generate Type II NB clones with sufficient Syp knockdown. However, both Type I and II NBs require Syp to express Eip93F (fig. S5, E and H). In both types, the null *syp* allele completely abolished Eip93F expression, while the hypomorphic *syp* allele was able to rescue it, suggesting a similar genetic interaction between *syp* and *Eip93F* in both Type I and II NBs.

Next, to investigate whether the RBPs also destabilize target mRNAs, we examined mutual regulation between Imp and Syp. While Imp and Syp genetically repress each other (*17*, *27*, *29*), the exact mechanism remains unclear. We used central brain NBs at 48 hours ALH (Fig. 1A), where they exhibit intrinsic heterogeneity of Imp and Syp protein levels, and correlated RNA transcription and half-life against the protein level of the other partner RBP. Notably, in both cases, transcription levels of *imp* and *syp* did not correlate with the opposing RBP’s protein (fig. S5, I to J), suggesting that post-transcriptional control may establish their gradients as NBs age (*17*). Here, *syp* RNA half-life negatively correlated with Imp protein levels (Fig. 5I), indicating that Imp destabilizes *syp* at high levels (early NB stages). On the other hand, a reciprocal regulatory correlation between Syp protein on *imp* RNA stability was not observed (Fig. 5J), implying distinct mechanisms or regulators involved with *imp*. Together, our results suggest that Imp and Syp are key post-transcriptional regulatory effectors of temporally expressed genes. We propose that many more transcripts that dynamically interact with these RBPs may contribute to specifying coarse or fine temporal windows.

### Competitive binding interplay between Imp versus Syp is rare

**Fig. 6.**
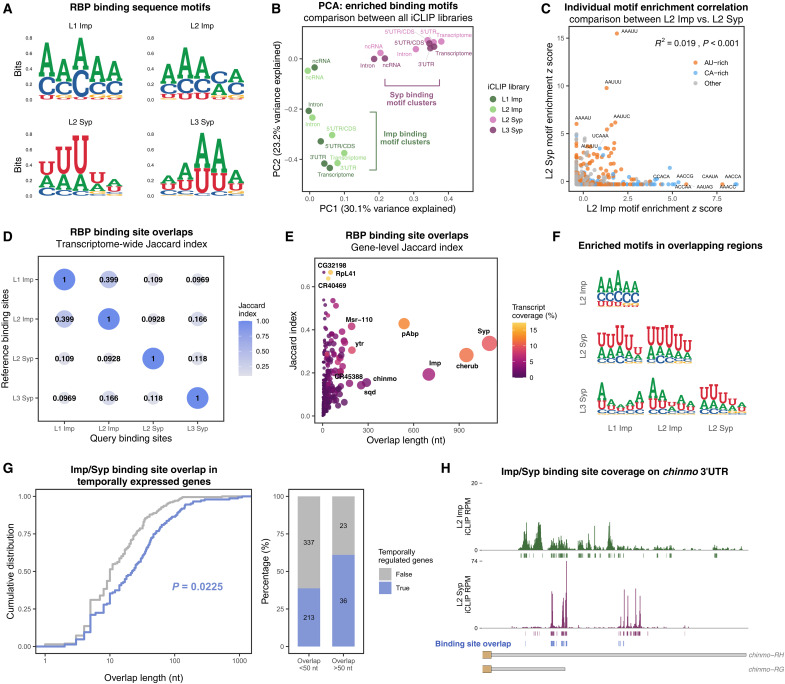
Imp and Syp binding sites poorly overlap, except in temporally expressed transcripts. (**A**) Enriched RNA sequence motifs for Imp and Syp binding sites, with nonsignificant cross-links (FDR > 0.01) used as backgrounds using positionally enriched *k*-mer analysis (PEKA; see Materials and Methods). Consensus motifs obtained across all transcriptomic regions are shown. (**B**) PCA of enrichment scores for all sequence motifs (5 to 8 *k*-mers) from Imp and Syp iCLIP libraries. Enrichment scores are further divided into transcriptomic regions. Note the separation of Imp and Syp libraries, while L1/L2 Imp and L2/L3 Syp motifs cluster closely with each other. (**C**) Correlation of individual sequence motif enrichment scores between L2 Imp and L2 Syp iCLIP libraries. AU-rich or CA-rich motifs are colored separately, and *R*^2^ correlation value is shown. (**D**) Transcriptome-wide overlap between Imp and Syp iCLIP binding sites. The degree of overlap was assessed using the Jaccard index method. (**E**) Gene-level overlap between L2 Imp and L2 Syp iCLIP binding sites. The size of the symbol represents the absolute nucleotide-length of the overlap. (**F**) Enriched RNA binding sequence motifs specifically in the overlapping binding sites between Imp and Syp iCLIP libraries. (**G**) Cumulative distribution comparing L2 Imp and L2 Syp iCLIP binding site overlap between temporally regulated and nontemporally regulated genes (left). Bar plot showing the group of temporally expressed genes and whether Imp and Syp binding sites overlap more than 50 nt on their mRNA. Temporally regulated genes were taken from staged NB and brain scRNA-seq datasets (*17*, *18*, *39*). (**H**) Imp and Syp iCLIP coverages on the *chinmo* 3′UTR region. Overlapping binding sites are highlighted in blue.

Distinct signatures of Imp and Syp occupancy on target transcripts indicate that their molecular interplay plays a crucial regulatory role. Therefore, we next investigated whether iCLIP peak distributions could reveal regulatory modalities of the interplay between Imp and Syp. First, we considered a scenario where Imp and Syp directly compete for the same binding sites. To address whether Imp and Syp can recognize similar RNA sequences, we performed a motif enrichment analysis by comparing iCLIP peaks versus background cross-links (*60*). As expected, given the 3′UTR preference, Syp binding sites were highly enriched in AU-containing motifs, potentially part of the regulatory AU-rich elements (Fig. 6A). Imp, at both stages however, predominantly bound to CA-rich motifs, although a small number of AU-rich motifs scored above the background (fig. S6A). Consistently, PCA of *k*-mer enrichment scores revealed a clear distinction between Imp and Syp binding motifs across mRNA and ncRNA features (Fig. 6B and fig. S6, A and B). We also examined individual motif enrichments at the L2 stage where both RBPs coexpress and spatially coincide, yet it revealed a poor correlation (*R*^2^ = 0.019) between Imp and Syp binding sites (Fig. 6C), indicating the lack of consensus sequence motifs that are commonly recognized.

To explore this further, we investigated the physical relationship between Imp and Syp footprints on their cotargets. At a global scale, we found a statistically significant proximity between Imp and Syp iCLIP peaks (fig. S6C). However, direct overlap measurements yielded very small Jaccard indices, suggesting poor likelihood of overlapping binding events (Fig. 6D). We repeated this analysis at the individual gene level and also found a generally weak overlap across most cotargets (Fig. 6E). However, we identified an outlier group of transcripts (<5%) with notable degrees of overlap, many of which are known to be regulated temporally, such as *chinmo*, *lncRNA:cherub*, and *sqd* as well as *imp* and *syp* themselves (Fig. 6E). Within these regions, AU-rich sequence motifs were more enriched over CA-rich motifs (Fig. 6F). We hypothesized that temporal genes under regulatory influence of Imp and Syp could experience direct competition between the two RBPs. To test this, we compared coverage of overlapping binding sites between temporal and nontemporal genes identified from scRNA-seq or mechanically dissociated NB transcriptomes (*17*, *18*, *38*, *39*). We found that temporal genes harbor longer regions of Imp and Syp footprint overlap than nontemporal genes (Fig. 6G). Of note, as illustrated with *chinmo*, the absolute extent of overlaps was still modest in most genes, where only 17% of the RBP coverages were coincidental (Fig. 6H). However, *syp* was a notable outlier with 35% coincidental footprints (Fig. 6E), which suggests that mutual post-transcriptional regulation between Imp and Syp could occur through these overlapping sequence regions (Fig. 5I). We propose that a rare subset of temporally regulated transcripts may experience direct competition between Imp and Syp for the same binding sites, although this competition is unlikely to be a globally deployed regulatory mode.

### Imp and Syp binding sites show coevolutionary and combinatorial binding signatures

**Fig. 7.**
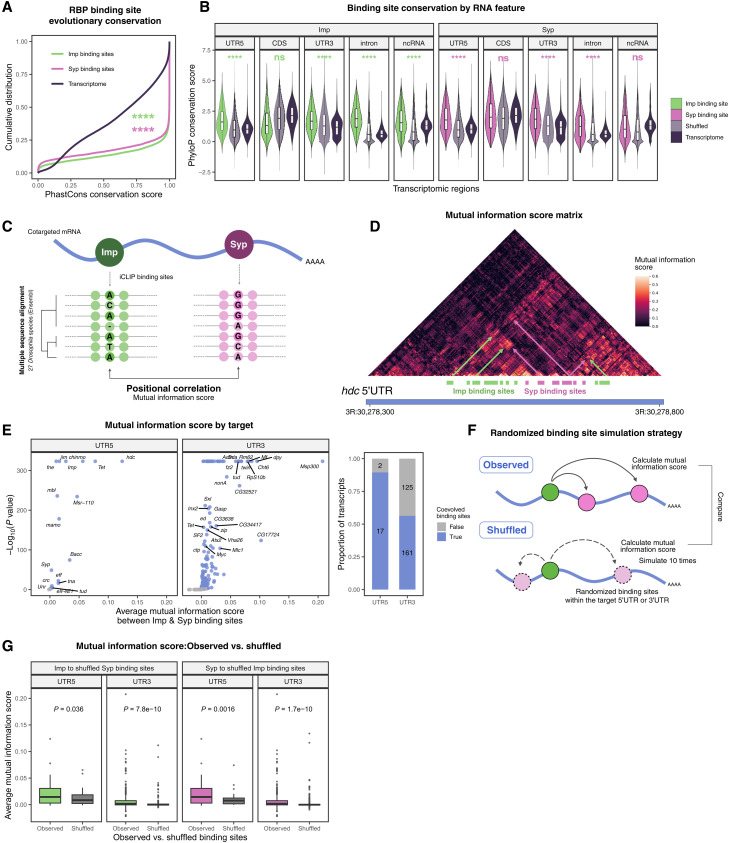
Imp and Syp binding sites display evolutionary linkage. (**A**) Comparison of PhastCons RNA sequence conservation score of Imp and Syp binding sites versus the larval brain transcriptome average. 27–way *Drosophila* PhastCons track was taken from the UCSC genome browser. ****P < 0.0001. (**B**) Analysis of PhyloP RNA sequence conservation score of Imp and Syp binding sites per gene and per transcript feature. The same scores calculated for the brain transcriptome and the shuffled iCLIP binding sites were used as controls. PhyloP was used instead of PhastCons due to its independence scoring between short-range nucleotide distances. For shuffled binding sites, the simulation was iterated 10 times per query. ****P < 0.0001. (**C**) A strategy to calculate mutual information (coevolution) score between Imp and Syp binding sites. To limit the analysis to confident binding sites, iCLIP peaks present in both L1/L2 for Imp and L2/L3 for Syp were used. (**D**) A correlation matrix of mutual information score between every nucleotide pair in the *headcase* (*hdc*) 5′UTR. Local clusters of high mutual information score coincide with Imp and Syp binding site pairs. (**E**) Average mutual information (coevolution) scores of Imp and Syp binding sites per gene. For each target gene, the evolutionary linkage was assessed by comparing average mutual information score between Imp and Syp binding site pairs versus their column average. Adjusted *P* value < 0.01 was considered significant. Because of computational memory constraints, mutual information calculations were subdivided between 5′UTR and 3′UTR. (**F**) Control simulation experiment for mutual information score calculation. The schematics illustrate a strategy to compare the distribution of average mutual information scores in observed (empirical iCLIP binding sites) versus randomized binding sites (shuffled). The simulation was performed 10 times per gene per UTR region and in both Imp-to-Syp and Syp-to-Imp directions. (**G**) Comparison of average mutual information scores in observed and shuffled settings, as described in (F).

To further characterize the functional importance of Imp and Syp binding to their target transcripts, we examined whether their binding sites are evolutionarily conserved. We calculated phastCons sequence conservation score (across 27 *Drosophila* species) (*61*) of iCLIP peaks and found that Imp and Syp binding regions were significantly more conserved than the brain transcriptome (Fig. 7A and fig. S7A). To rule out targeting bias toward conserved genes, we calculated average phyloP sequence conservation scores for each transcript feature in each gene and compared these with shuffled RBP binding sites per queried region. We found that Imp and Syp binding sites were highly conserved across multiple transcript features, except for CDS in Imp and ncRNA in Syp (Fig. 7B). Similar conservation trends were also observed when expanding the analysis to include 124 insect species (fig. S7, A to D), indicating a functional importance of Imp and Syp binding in maintaining evolutionary fitness.

Expanding on the RBP footprint conservation, we next explored whether Imp and Syp binding sites jointly mutated to maintain genetic interactions. Given the limited overlap between Imp and Syp binding sites, we hypothesized that their combinatorial mode of molecular interplay could be detected through evolutionary couplings (*62*). To investigate this, we performed mutual information analysis using a phylogenetic inference across 27 *Drosophila* species to identify pairs of coevolving nucleotides within target mRNA sequences (Fig. 7C) (*63*–*65*). We calculated mutual information scores across 5′UTR or 3′UTR of the shared RNA targets of Imp and Syp at the L2 stage where the RBPs spatially coexpress (full analysis output is available in data S4). We found local clusters of high-scoring nucleotide pairs that frequently coincided with Imp and Syp binding site pairs [illustrated with the *headcase* (*hdc*) 5′UTR score matrix; Fig. 7D], which is suggestive of coevolutionary linkage between their binding sites. Globally, we observed significantly higher average mutual information scores between Imp and Syp footprint pairs compared to individual row averages in 89 and 56% of the 5′UTR and 3′UTR targets, respectively (Fig. 7E). To reinforce our finding, we performed a control simulation experiment. We calculated coevolution scores upon randomized shuffling of the partner RBP binding sites and simulated this 10 times for both Imp-to-Syp and Syp-to-Imp directions (Fig. 7F). In this scenario, the significantly lower average mutual information scores in the shuffled simulations compared to the experimentally observed values further supported the pairwise evolutionary interaction between Imp and Syp footprints (Fig. 7G). Overall, our results show that Imp and Syp binding sites are highly conserved and likely jointly mutated to maintain their regulatory link. Therefore, we propose that combinatorial interplay, rather than competitive binding, between Imp and Syp could be more widespread in producing correct regulatory outcomes for their downstream cotargets.

## DISCUSSION

Here, we identify transcriptome-wide targets of Imp and Syp, a pair of RBPs that exhibits the most dynamic temporal expression gradient during the postembryonic brain development. RBPs control gene expression by regulating the RNA metabolism of their targets, making the elucidation of their RNA interactomes crucial for understanding their core functionalities. Temporal patterning in larval and pupal stages requires a combination of TFs and the opposing gradients of Imp and Syp (*14*, *17*, *21*, *29*). More than 60% of VNC neurons are born during this period, with their initial patterning at this stage preconfiguring adult neuronal identity (*56*). Much work has been done to understand the chromatin-level control of temporal specification. However, discordant transcription and translation activity of terminal differentiation genes (e.g., neurotransmitters) suggest that RNA regulation can also play a substantial role (*66*). Our high-resolution study of RBP target landscapes and their RNA footprints contributes to understanding the connections between concurrent transcriptional and post-transcriptional mechanisms shaping the nervous system.

Opposing Imp and Syp temporal gradients have been described to pattern multiple postembryonic lineages in the central brain, VNC, and optic lobe (*14*, *17*, *18*, *23*). While the slopes of Imp and Syp expression vary considerably between lineages, it is likely that the RBP gradient assigns coarse fates in these lineages, with temporal TF cascades downstream of Imp and Syp further diversifying neuronal subtypes (*19*). Our RBP occupancy analysis highlights groups of transcripts, enriched for TFs and RBPs, with distinct profiles of temporal Imp and Syp relative occupancy (Fig. 4, A and B). These transcripts are good candidates for defining fine temporal subwindows as differential RBP occupancy may lead to different regulatory outcomes. Consistently, our results show that Imp and Syp can post-transcriptionally regulate the expression of temporally controlled transcripts (Fig. 4). Future studies on their targets will be valuable for understanding how neuronal subidentities are generated and which mechanisms, such as programmed cell death, ensure correct proportions of neuron types. In other respects, unique modes of division deployed in Type II intermediate neural progenitor (INP) and optic lobe lineages indicate that spatial patterning mechanisms act concurrently with temporal gradients to diversify neural fates (*12*, *67*, *68*). While this study aimed to identify brain-wide targets of Imp and Syp, identifying cell type–specific RNA interactomes may facilitate future studies on the cooperation between multiple patterning mechanisms. For example, the HyperTRIBE technique, which uses UAS-RBP::ADAR fusion constructs, will be useful to decipher lineage or cell type–specific RBP targets (*69*).

Our dataset captures Imp and Syp targets involved in diverse biological and molecular functions, including key regulators of NSC growth and quiescence, energy metabolism, intracellular signaling, hormone response, and tumorigenic potential. This indicates the versatility of Imp and Syp gradients in mediating intrinsic NB lineage functions as well as responding to extrinsic signals (*14*, *19*, *36*, *37*). Notably, both Imp and Syp bind to many transcripts encoding cytoskeleton and axonogenesis regulators (Fig. 2, A and B). Despite their known roles in synaptic transmission and cytoskeletal remodeling, the developmental context of Imp and Syp in circuit connectivity is not well understood (*31*, *32*, *34*). In the central brain Type I lineage, early-born neurons form extensive neurite projections, while late-born neurons display decreasing morphological complexity (*70*). Both Imp and Syp proteins localize to nerve bundles and distal tips of immature neurites; therefore, it would be interesting to investigate the regulatory function of Imp and Syp within these fine neuronal projections.

Given their predominantly cytoplasmic localization, Imp and Syp likely regulate the stability, translation, and/or localization of their downstream target transcripts (*71*). This is reflected in the enrichment of iCLIP binding sites in the 5′ and 3′UTRs, which harbor cis-regulatory sequences that may respond to changing Imp and Syp levels (*35*, *72*). A key example is *chinmo*, where Imp promotes translation while Syp represses translation, resulting in a steep gradient of the protein product despite a relatively constant level of its mRNA (*17*). The substantial overlap between Imp and Syp RNA interactomes suggests that they may influence divergent expression of many more genes in the brain. Therefore, it is important to consider the mechanistic interplay between trans-acting factors as a critical layer of regulation (*73*). For example, regulatory interplay between RBPs can be competitive when they occupy overlapping binding sites, causing steric hindrance (*74*). However, our RBP profiling method does not support this as the primary mode of interplay, as Imp and Syp recognize distinct sequence motifs with limited binding site overlaps (Fig. 6). Nevertheless, these small overlapping regions might still act as key regulatory hubs for direct competition and regulatory outcomes (*75*). Future mutagenesis studies are needed to determine whether these overlapping binding sites are essential for the transcript’s response to the opposing gradients of Imp and Syp.

Alternatively, RBPs can bind to the same RNA in a combinatorial manner, where differential occupancy can have synergistic or antagonistic effects on target gene expression (*73*). This mode of interplay also includes RNA-dependent interactions, such as binding of one RBP triggering allosteric changes in the RNA structure that facilitates or hinders the binding of another RBP (*76*). Our binding site coevolution analysis supports the combinatorial interplay between Imp and Syp and the conservation of the Imp-Syp regulatory cassette in many cotargeted mRNAs (Fig. 7). In this context, Imp and Syp likely regulate the fate of the bound mRNA by recruiting other effector proteins or forming processive multi-RBP complexes. In mammals, IGF2BP1 associate with HNRNPU, SYNCRIP, Y box binding protein (YBX1), and DExH-box helicase 9 (DHX9) to control *c-myc* stability, and HNRNPQ has been shown to interact with poly(A)-binding protein (PABP) to regulate cap- and internal ribosomal entry site (IRES)–mediated translations (*77*–*79*). Consistent with this idea, our dataset shows overrepresentation of transcripts encoding RBPs targeted by Imp and Syp. This suggests that Imp and Syp could influence expression of cointeracting partner RBPs to tailor their post-transcriptional regulatory niche. It would be important to elucidate protein interactors of Imp and Syp in the brain and how combinatorial binding of Imp and Syp affects recruitment of these factors. Future RBP profiling experiments in larval tissues, similar to this study, will be valuable to investigate binding site co-occurrences across RBPs and discover previously unidentified cointeracting RBPs.

Mammalian homologs of Imp and Syp are also expressed in the developing brain and play crucial roles in neuro/synaptogenesis (*80*, *81*). For example, in mice, IMP1 levels rapidly decline in a temporal fashion to regulate maintenance and differentiation of vertebrate NSCs (*82*). Likewise, *snRNA:7SK*, a high target of fly Syp, has been shown to interact with hnRNPR to regulate local transcriptome in neurites (*48*). In the current study, we found an unexpected degree of evolutionary conservation in RNA target preference and RNA sequence recognition between invertebrate and vertebrate orthologs of Imp and Syp (data S1). Morphogenetic gradients and temporal cascade of RBPs and TFs are likely conserved in mammalian neurogenesis to specify young and old cellular fates (*9*, *83*–*85*). In the future, it will be interesting to determine whether orthologous temporal factors downstream of Imp and Syp play conserved roles in the developing vertebrate CNS.

## MATERIALS AND METHODS

### Fly genetics

All fly stocks were raised on standard cornmeal-agar medium at 25°C. The following genotypes were used: *Oregon-R* (wild type, RRID_BDSC:5), *Imp::GFSTF* (RRID_BDSC:60237), *Syp::GFP* (*34*), *insc-GAL4* (gift from A. Baena-Lopez), *UAS-syp RNAi* [Vienna Drosophila Resource Center (VDRC), 33011], *tub-GAL80ts; TM2/TM6B* (RRID_BDSC:7108), *UAS-imp RNAi* (RRID_BDSC:34977), *UAS-blank RNAi* (empty wild-type control; VDRC, 60100), *syp^f03775^* (*syp* hypomorphic allele, RRID_BDSC:85668), *syp^e00286^* (*syp* null allele, RRID_BDSC:95448), *Df(3R)BSC124/TM6B* (*syp* deficiency, RRID_BDSC:9289), *hsFLP; act5c>Flipout>GAL4, UAS-GFP* (gift from A. Baena-Lopez), and *PntP1-GAL4* (Type II NB lineage driver, gift from Y. Jan). For iCLIP experiments, male and female larvae were developmentally synchronized at the larval-hatching stage by collecting newly hatched flies within a 2-hour window into apple juice plates supplemented with yeast paste. For experiments using NB clonal analysis, GAL4-positive NB clones were generated using a heat-shock inducible flippase (*hsflp*) element and a GAL4 flip-out cassette under the control of a transcription stop sequence flanked by Flippase Recombinase target (FRT) sites. Appropriately staged larvae were heat-shocked at 37°C for 15 to 30 min in a water bath to express *hsflp*, which stochastically induces GAL4-positive NB clones. The heat-shocked flies were maintained at 29°C until dissection.

### Antibodies

The following antibodies were used: guinea pig anti-Syp (*43*) [Western blot (WB), 1:1000; Immunoprecipitation (IP), 1:100; Immunofluorescence (IF), 1:500], rabbit anti-Imp (gift from P. MacDonald; IF, 1:500), guinea pig anti-Eip93F (gift from C. Doe; IF, 1:500), rat anti-Dpn (Abcam, ab195173; RRID:AB_2687586; IF, 1:200), mouse anti-Lamin [Developmental Studies Hybridoma Bank (DSHB), ADL67.10; RRID:AB_528336; IF, 1:10], mouse antipuromycin (Sigma-Aldrich, ZMS1016; RRID:AB_3099686; IF, 1:50), rat anti-GFP (Chromotek, 3H9; RRID:AB_10773374; WB, 1:1000), and mouse anti-FLAG M2 (Sigma-Aldrich, F1804; RRID:AB_262044; WB, 1:1000). Alexa Fluor-conjugated secondary antibodies (Thermo Fisher Scientific) were used at 1:500 for immunofluorescence. IRDye-conjugated secondary antibodies (LI-COR) were used at 1:10,000 for immunoblotting.

### iCLIP library preparation

For whole-larval and larval-head iCLIP, samples were collected and snap-frozen in liquid nitrogen. Frozen biosamples were ground into fine powder in a liquid nitrogen–cooled mortar and a pestle and were subjected to 254-nm UV-C irradiation four times at 150 to 200 mJ/cm^2^ using a Stratalinker 2400 (Stratagene). For larval brain iCLIP, wandering L3 brains (96 hours ALH) were dissected in cold phosphate-buffered saline (PBS) supplemented with 0.2 mM 4-2-aminoethyl)benzenesulfonyl fluoride hydrochloride (AEBSF) in batches of 50 brains, and UV-C was irradiated four times at 150 mJ/cm^2^ with shaking in-between. Cross-linked samples were lysed in CLIP lysis buffer (*42*) supplemented with 0.2 to 2mM AEBSF (Sigma-Aldrich), 1 to 2× complete protease inhibitor (Roche), 1 mM ribonucleoside vanadyl complex [RVC; New England Biolabs (NEB)], and RNAsin plus (40 U/ml; Promega). Lysates were sonicated using a Bioruptor (Diagenode) with 10 cycles of 30-s on/off periods at low-intensity setting. Lysates were cleared by centrifugation and filtration using Co-star 0.22-μm spin filters (Sigma-Aldrich), and protein levels were quantified using the Pierce 660 Protein Assay (Thermo Fisher Scientific). Quantified lysates were normalized to 0.5 to 1.5 mg/ml, snap-frozen, and stored at −80°C.

iCLIP sequencing libraries were generated using the iCLIP2 protocol with several modifications from the infrared CLIP and the enhanced CLIP (eCLIP) protocols (*42*, *44*, *45*). Cross-linked RNAs in the lysates were partially trimmed ribonuclease I (RNAse I; 0.15 to 0.5 U/μl; Ambion) for 3 min at 37°C. GFP-tagged Syp (L3 brains) was immunoprecipitated using GFP-Trap Magnetic Agarose Beads (Chromotek, gtma-20), and Syp (L2 heads) was immunoprecipitated using guinea-pig anti-Syp (*43*) coupled to Dynabeads Protein A (Invitrogen). FLAG-tagged Imp was pulled down with Magnetic M2 FLAG Beads (Sigma-Aldrich, M8823). Immunoprecipitations and washes were performed as described in the iCLIP2 protocol, and the RNAs were 3′end dephosphorylated by T4 Polynucleotide Kinase (NEB) and FastAP phosphatases (Thermo Fisher Scientific). Then, infrared IR800 dye–conjugated 3′ adapters were ligated overnight using RNA ligase 1 (NEB) in a high-percentage polyethylene glycol 8000 buffer. Molecular weight distribution of the protein-RNA complexes was visualized after 3 to 8% tris-acetate SDS–polyacrylamide gel electrophoresis (SDS-PAGE) and transferred to nitrocellulose membranes, and then appropriate size regions (~75 kDa above the expected molecular weight of the RBPs) were excised to extract RNA using proteinase K (Roche) in an SDS-containing buffer. The RNAs were further purified with phenol/chloroform/isoamyl alcohol mix (pH 6.6) followed by clean-up with the RNA Clean and Concentrator Kit (Zymo Research). The purified RNAs were reverse-transcribed using SuperScript IV (Invitrogen), and the cDNA was purified with MyONE Silane beads (Thermo Fisher Scientific). Second DNA adapters containing unique molecular identifiers (UMIs) and experimental barcodes were attached to the 3′end of the cDNAs overnight using RNA ligase 1, and the reaction clean-up was performed using MyONE Silane beads. The cDNAs now flanked by Illumina adapters at both ends were amplified using a two-step polymerase chain reaction (PCR) strategy. First, cDNAs were amplified with 6 PCR cycles using short Solexa P3/5 primers to allow bead-based size selection with ProNex Beads Size-selection Chemistry (Promega). To avoid overamplification, the optimal number of cycles for the second amplification was calculated using 1 μl of the first PCR product from quantitative PCR (qPCR) Ct values. Both PCR and qPCR were performed using Pfusion High-Fidelity Polymerase Master Mix (NEB) and full-length Solexa P3/5 primers, and Evagreen (Biotium) was added as a reporter dye in qPCR reactions. After the second PCR amplification, libraries were subjected to a second round of size selection by 2.4× ProNex Chemistry to deplete excess primers and molecules shorter than 150 bp.

SMInput libraries were prepared in parallel with immunoprecipitated samples. Ten microliters of the RNAse I–digested lysates was separated via SDS-PAGE alongside the paired immunoprecipitation sample. After transfer to a nitrocellulose membrane, the same molecular weight region was excised, hence size-matched, and RNA was extracted, which represents the background RBP cross-link–ome. Purified RNAs were 3′end dephosphorylated and adapter-ligated in solution followed by MyONE Silane clean-up after each step. Subsequently, the SMInput RNAs were processed alongside the immunoprecipitation sample at the reverse transcription stage.

Normalized and pooled libraries were sequenced using a NextSeq 500 sequencer (Illumina) at the Department of Zoology, University of Oxford. High-output cartridge v2.5 was used for 92 cycles in single-end mode.

### Bioinformatic methods

#### iCLIP computational analysis

Raw FASTQ files from iCLIP were preprocessed as described in (*86*) with minor modifications. The reads were mapped to the Ensembl *D. melanogaster* genome assembly version 99 (BDGP6.28) using STAR with end-to-end mapping (https://github.com/alexdobin/STAR). PCR duplicated cDNAs were filtered on the basis of UMI sequences and mapping locations using UMI tools (https://github.com/CGATOxford/UMI-tools). Cross-link sites were extracted, which were defined as the nucleotide position preceding the cDNA insert (i.e., one nucleotide before the reverse transcription truncation events). Significant (FDR < 0.01) cross-link sites and RBP binding peaks were called using the iCount package (https://icount.readthedocs.io) with an FDR threshold of 0.01 (*87*). The binding peaks were further filtered by comparing iCLIP versus SMInput libraries using Differential Expressed Windows-seq (DEW-seq) (*88*, *89*) with log_2_ fold change > 1 and adjusted *P* value < 0.05 threshold, which controls background noise and false-positive sites. One of the L3 Syp SMInput libraries was discarded from the analysis due to limited usable sequencing depth. DEW-seq, which uses the DESeq2 algorithm underneath, is capable of accounting for differences in replicate numbers. Lastly, significant and enriched binding sites were normalized to 5-nt length using the R script provided in (*86*).

#### RNA-seq analysis

Public RNA-seq datasets were reanalyzed using the Kallisto-DESeq2 method (https://pachterlab.github.io/kallisto/). Raw FASTQ files were downloaded from Gene Expression Omnibus (GEO) and were filtered for fly ribosomal RNA and low-complexity reads using BBDuk (https://github.com/BioInfoTools/BBMap). Filtered reads were pseudoaligned to the Ensembl *D. melanogaster* transcriptome assembly version 99 using Kallisto. The custom transcriptome index was created using Kallisto-Bustools to include flanking intron sequences (25 nt) at either ends of exons. Estimated counts and transcripts per million (TPM) for transcript isoforms were collapsed for each gene. Differential expression analysis was performed using the R package DESeq2. The normalization for library size was performed within the DESeq2 analysis, and multiple hypothesis testing was done via the Independent Hypothesis Weighting (IHW) method implemented in the R package IHW. For differential expression of genes in NB temporal datasets (*17*, *18*), the pseudoaligned counts were processed using maSigPro with default settings.

#### Area under recovery curve cell measurements

scRNA-seq gene expression matrices and associated embedding information were downloaded from Scope (https://scope.aertslab.org/) or from (*39*). To identify cells with active gene sets (expression of Imp and Syp target transcripts), area under recovery curve cell (AUCell) was used (https://github.com/aertslab/AUCell). For each cell from the scRNA-seq dataset, AUCell scores for combined Imp targets (L1 + L2) and Syp targets (L2 + L3) were calculated from non-normalized gene expression matrices. The resulting scores represent the proportion of expressed transcripts in the query gene set and their relative expression value compared to other transcripts in the cell.

#### Functional annotation and enrichment analysis

GO, Reactome, and KEGG pathway enrichment analyses were conducted using the R package clusterProfiler. Terms that are log_2_ fold change > 1 and adjusted *P* value < 0.05 were considered significantly enriched. For “Biological Process” GO terms, similar terms were grouped using the “binary_cut” algorithm using the R package GOSemSim. For “Molecular Function” GO terms, genes were grouped into GOSlim terms represented in FlyBase gene function ribbon.

#### RBP target comparison between species

Human IMP1-3 eCLIP data from pluripotent stem cells were filtered for cross-link fold change > 2 to identify IMP targets (*47*). Rat SYNCRIP iCLIP targets from primary neurons and mouse hnRNPR iCLIP targets from primary motoneurons and NSC34 (motoneuron) cell line were downloaded from following publications (*48*, *49*). Human and murine genes were converted to fly genes using the DIOPT Application Programming Interface (API), and only high-confidence orthologs (DIOPT score ≥ 8) were retained for comparisons.

#### RBP binding sequence motif discovery

Enriched RBP binding sequence motifs were identified using the positionally enriched *k*-mer analysis (PEKA) tool (*60*). Significant cross-links (identified from iCount, FDR < 0.01) that are also enriched over SMInput libraries were used as high-confidence thresholded cross-links (tXn). Immunoprecipitation-recovered cross-links (*iCount xlsite* output) were used as reference background cross-links (oXn). Default PEKA settings were used for the analysis with no genome masking. For both Imp and Syp, the 5-mer analysis yielded the highest enrichment scores among the 4- to 8-mers tested. Consensus motifs were plotted using the R package motifStack.

#### Genome interval correlation methods

Relative distances and overlaps (Jaccard indices) between Imp and Syp iCLIP peaks were analyzed using the R packages GenometriCorr and valr. For the relative distance metric, the presented correlation index can range from −1 to +1 where the value of −1 indicates perfectly even spacing between binding sites. However, values closer to +1 indicate closer proximity between genomic intervals.

#### Coevolution analysis

Evolutionary coupling between Imp and Syp binding sites was assessed using the mutual information positional correlations (*62*). The pairwise mutual information was calculated as described in (*63*–*65*), and scripts from the Hilgers Lab Zenodo repository were used (https://zenodo.org/records/7759440). Briefly, multiple sequence alignment track of 27 *Drosophila* species was downloaded from the UCSC genome browser and filtered using “refineMSA()” function in the Python package ProDy (http://prody.csb.pitt.edu/) to keep sequences with 60% gaps (rowocc = 0.4) and 98% identity level (seqid = 0.98). Nucleotide-level pairwise mutual information scores were normalized using the average product correction method using ProDy. For each target transcript of Imp and Syp, average mutual information scores were compared between Imp and Syp binding site pairs versus the Imp or Syp binding site columnar average. The comparison was performed in both Imp-to-Syp and Syp-to-Imp directions. For high confidence binding sites, the analysis was limited to Imp and Syp iCLIP peaks that occur in both developmental time points. Because of computational memory constraints, 5′UTR and 3′UTR portions of the mRNA were computed separately. The resulting mutual information score indicates probability to estimate whether a given nucleotide change will be accompanied by another nucleotide change. For control simulations, shuffled Imp or Syp binding sites for each transcript were generated using “bed_shuffle()” function in the R package valr.

#### Hierarchical clustering of RBP binding interactions

To identify groups of transcripts that differentially interact with Imp and Syp across development, we first removed background reads from iCLIP libraries by subtracting average background read counts from nonsignificant (FDR > 0.01) cross-links obtained from iCount peaks output. For each gene, significant iCLIP reads (FDR < 0.01) were then converted to reads per million (RPM), normalized to gene expression levels (TPM) in the larval brain, and averaged across three biological replicates. We performed *k*-means clustering of normalized iCLIP RPM using the R package MaSigPro with following parameters: significance threshold (*Q*) = 0.01, negative binomial theta = 10, *R*-squared regression fit threshold = 0.7, and clustering method = hierarchical clustering (hclust), *k* = 6. For plotting purposes only, minimal pseudo-counts were added to genes where no iCLIP read counts were recovered above background.

### Microscopy methods

#### RNA smFISH

For smFISH, tissues were dissected in PBS or Schneider’s medium, rinsed once with PBS, and fixed with 4% paraformaldehyde diluted in PBS/0.1% Triton X-100 (PBSTx) for 25 min at room temperature (RT). Samples were rinsed briefly in PBSTx and then further permeabilized with PBSTx twice for 20 min each. Samples were prehybridized in smFISH wash solution (10% formamide, 2× SSC) for 30 min at 37°C and hybridized in smFISH hybridization solution (10% formamide, 2× SSC, 10% dextran sulphate) with probes diluted to 250 nM final concentrations overnight at 37°C. Samples were rinsed briefly in smFISH wash solution and then washed in smFISH wash solution twice for 30 min each at 37°C. Last, samples were washed in 2× SSC for 10 min at RT and mounted on slides using either Vectashield (Vector labs) or Slowfade Diamond (Thermo Fisher Scientific) antifade mounting media. Slides were either imaged immediately or stored at 4°C. Counterstains were included in the wash solution: 4′,6-diamidino-2-phenylindole (DAPI; 1 μg/ml) and Alexa Fluor 488–conjugated phalloidin (1:100; Thermo Fisher Scientific, A12379). smFISH probe sequences used in this study are available in data S5.

#### smFISH probe synthesis

Candidate smFISH probe sequences were generated using the Stellaris Probe Designer version 4.2 (https://biosearchtech.com/stellaris-designer) with the following parameters: “masking level, 5; oligo length, 20 nt; minimum spacing length, 3 nt.” Oligonucleotides were singly labeled with ATTO 488 (ATTO-Tec), ATTO 633, Alexa Fluor 647 (Thermo Fisher Scientific), or Cyanine 3 (Lumiprobe) at the 3′ ends according to a published protocol (*90*). Dye-labeled oligos were purified using the Oligo Clean and Concentrator Kit (Zymo Research), and the probe concentrations were normalized to 25 μM using NanoDrop measurements. All probe sets used in this study had a degree of labeling > 0.96.

#### Immunofluorescence

Tissues were dissected and fixed as with smFISH procedure. After permeabilization, samples were blocked in blocking solution (LiCor Odyssey blocking buffer supplemented with 0.1% Tween 20) for 30 to 60 min at RT. Then, samples were incubated with primary antibodies diluted in blocking solution. Samples were probed overnight at 4°C and were washed three times in PBS/0.1% Tween 20 (PBSTw) for 10 min each at RT and incubated with fluorescent secondary antibodies (1:500) diluted in blocking solution overnight at 4°C. After further three washes in PBSTw, cells were mounted in an antifade mounting medium. Antibodies used in this study are listed in the resource table. For combined smFISH and immunofluorescence, antibody staining was carried out sequentially after the smFISH protocol. In addition, the blocking solution was pretreated with 1:25 RNASecure (Invitrogen) for 30 min at 50°C and supplemented with 2 mM RVC to prevent RNase activity.

#### Puromycin incorporation assay

Dissected L3 larval brains were washed briefly in brain culture media [80% Schneider’s medium, 20% fetal bovine serum, and insulin (100 μg/ml)] and incubated in brain culture media with or without puromycin (5 μg/ml). Tissues were incubated ex vivo for 1 hour at RT with shaking at 100 rpm. For control experiments, cycloheximide treatment (100 μg/ml) was added at the same time with puromycin. Incorporated puromycin was visualized using anti-puromycin (Sigma-Aldrich, ZMS1016) via immunofluorescence. Only the top layer of central brain NB lineages was imaged to ensure an even degree of puromycin penetration between tissues.

#### Image acquisition and handling

Mounted specimens were imaged on an Olympus SpinSR10 spinning disk confocal system equipped with a Prime BSI camera or on an Olympus FV3000 Inverted Laser Scanning Microscope. Objectives used were 20x dry [0.8 numerical aperture (NA), UPLXAPO20X], 60x silicone oil (1.3 NA, UPLSAPO60XS2), or 100x oil (1.45 NA, UPLXAPO100XO). Image voxel sizes were 0.55 μm by 0.55 μm by 2 μm (*x*:*y*:*z*) with the 20x objective and 0.11 μm by 0.11 μm by 0.2 μm (*x*:*y*:*z*) with the 60x and 100x objectives. Microscopes were driven using Olympus cellSens Dimension or FV313S software. Images were uploaded and stored in the University of Oxford OMERO server, and OMERO.figure was used to generate image visualizations.

#### Fluorescence intensity quantification

Immunofluorescence images were background-subtracted using the rolling ball subtraction method (radius = 50 to 150 pixels) in ImageJ. Antibody stain was quantified by integrating fluorescence intensity across the z-stacks of region of interest divided by the volume to obtain signal density. Camera background signals (area with no tissue) were subtracted from the signal density, and the resulting values were normalized to control conditions.

#### smFISH image quantification

smFISH images were quantified using a custom Python pipeline using Bigfish, Sci-kit image, and Numpy libraries. Briefly, exonic smFISH channels were background-subtracted with the skimage.white_tophat algorithm (radius = 5 pixels) and Laplacian of Gaussian (LoG) filtered with a theoretical point spread function based on microscope acquisition settings. Thresholds for spot detection were set manually on the basis of the intensity of LoG-filtered spots, and reference single-molecule images were obtained using the build_reference_spot() function iterating over the entire image dataset in Bigfish. Intranuclear transcription sites were localized using intronic smFISH channels, and corresponding exonic channels were used to resolve the number of nascent RNA using the decompose_cluster() function in Bigfish. For quantifying mRNAs per cell level, individual nuclei from the DAPI channel were segmented using the Cellpose (v0.7.2) “nuclei” model, and resulting masks were dilated two iterations using skimage Python package.

#### RNA half-life calculation

RNA half-lives were calculated using the TransQuant steady-state methodology (*57*). A MATLAB-to-R translated version of TransQuant script was used to obtain the probe library weighting factor, which accounts for the positional information of each oligonucleotide against the target transcript. Transcription rate, decay rate, and half-lives were calculated using the equations listed below. An elongation rate of 90 kb/hour was used for the calculation (*27*). For *Eip93F*, chromosome fraction was 2. For *jim* and *Ldh*, the proportion of cells positive for intronic smFISH signal per field of view was used as chromosome fraction.

1) Transcription rate (mRNA*hour^−1^) = [(nascent transcript number/weighting factor)*elongation rate]/gene length

2) Decay rate (hour^−1^) = (chromosome fraction *x* transcription rate *x* number of chromosome copies)/transcripts in the cell

3) Half-life (min) = [ln (2)/decay rate] * 60

#### Statistics and data presentation

All statistical analyses were performed using the R package rstatix. Data normality was assessed using the Shapiro-Wilk test. Comparisons were performed using either the parametric tests, *t* test and analysis of variance (ANOVA) test, or nonparametric tests, Mann-Whitney *U* or Dunnett’s test. Comparison of distributions was performed using the Kolmogorov-Smirnov test, as indicated in figure legends. Where applicable, *P* values were adjusted using Bonferroni’s method for multiple comparisons. For data wrangling, the tidyverse suite of packages was used in the RStudio environment, while Numpy and Pandas Python libraries were used in the Jupyter notebook environment. The R packages ggplot2, ggbeeswarm, scales, and patchwork were used to create the presented visualizations. Further visual annotations were made in Affinity Designer (Serif).

## References

[1] L.-J. Pilaz, D. L. Silver, Post-transcriptional regulation in corticogenesis: How RNA-binding proteins help build the brain. Wiley Interdiscip. Rev. RNA 6, 501–515 (2015). 26088328 10.1002/wrna.1289 PMC4624281

[2] M. Yano, Y. Hayakawa-Yano, H. Okano, RNA regulation went wrong in neurodevelopmental disorders: The example of Msi/Elavl RNA binding proteins. Int. J. Dev. Neurosci. 55, 124–130 (2016). 26796049 10.1016/j.ijdevneu.2016.01.002

[3] J. B. Skeath, S. Thor, Genetic control of *Drosophila* nerve cord development. Curr. Opin. Neurobiol. 13, 8–15 (2003). 12593977 10.1016/s0959-4388(03)00007-2

[4] C. Q. Doe, Neural stem cells: Balancing self-renewal with differentiation. Development 135, 1575–1587 (2008). 18356248 10.1242/dev.014977

[5] C. C. Homem, J. A. Knoblich, *Drosophila* neuroblasts: A model for stem cell biology. Development 139, 4297–4310 (2012). 23132240 10.1242/dev.080515

[6] E. Caussinus, F. Hirth, Asymmetric stem cell division in development and cancer. Prog. Mol. Subcell. Biol. 45, 205–225 (2007). 17585502 10.1007/978-3-540-69161-7_9

[7] C. C. F. Homem, M. Repic, J. A. Knoblich, Proliferation control in neural stem and progenitor cells. Nat. Rev. Neurosci. 16, 647–659 (2015). 26420377 10.1038/nrn4021 PMC4667397

[8] C. Maurange, A. P. Gould, Brainy but not too brainy: Starting and stopping neuroblast divisions in Drosophila. Trends Neurosci. 28, 30–36 (2005). 15626494 10.1016/j.tins.2004.10.009

[9] C. Q. Doe, Temporal Patterning in the Drosophila CNS. Annu. Rev. Cell. Dev. Biol. 33, 219–240 (2017). 28992439 10.1146/annurev-cellbio-111315-125210

[10] C. Q. Doe, Chinmo and neuroblast temporal identity. Cell 127, 254–256 (2006). 17055425 10.1016/j.cell.2006.10.008

[11] T. Isshiki, B. Pearson, S. Holbrook, C. Q. Doe, *Drosophila* neuroblasts sequentially express transcription factors which specify the temporal identity of their neuronal progeny. Cell 106, 511–521 (2001). 11525736 10.1016/s0092-8674(01)00465-2

[12] N. Konstantinides, I. Holguera, A. M. Rossi, A. Escobar, L. Dudragne, Y.-C. Chen, T. N. Tran, A. M. Martínez Jaimes, M. N. Özel, F. Simon, Z. Shao, N. M. Tsankova, J. F. Fullard, U. Walldorf, P. Roussos, C. Desplan, A complete temporal transcription factor series in the fly visual system. Nature 604, 316–322 (2022). 35388222 10.1038/s41586-022-04564-w PMC9074256

[13] C. Maurange, L. Cheng, A. P. Gould, Temporal transcription factors and their targets schedule the end of neural proliferation in *Drosophila*. Cell 133, 891–902 (2008). 18510932 10.1016/j.cell.2008.03.034

[14] M. H. Syed, B. Mark, C. Q. Doe, Steroid hormone induction of temporal gene expression in *Drosophila* brain neuroblasts generates neuronal and glial diversity. eLife 6, e26287 (2017). 28394252 10.7554/eLife.26287 PMC5403213

[15] A. Kepecs, G. Fishell, Interneuron cell types are fit to function. Nature 505, 318–326 (2014). 24429630 10.1038/nature12983 PMC4349583

[16] A. Franks, E. Airoldi, N. Slavov, Post-transcriptional regulation across human tissues. PLOS Comput. Biol. 13, e1005535 (2017). 28481885 10.1371/journal.pcbi.1005535 PMC5440056

[17] Z. Liu, C.-P. Yang, K. Sugino, C.-C. Fu, L.-Y. Liu, X. Yao, L. P. Lee, T. Lee, Opposing intrinsic temporal gradients guide neural stem cell production of varied neuronal fates. Science 350, 317–320 (2015). 26472907 10.1126/science.aad1886

[18] Q. Ren, C.-P. Yang, Z. Liu, K. Sugino, K. Mok, Y. He, M. Ito, A. Nern, H. Otsuna, T. Lee, Stem cell-intrinsic, seven-up-triggered temporal factor gradients diversify intermediate neural progenitors. Curr. Biol. 27, 1303–1313 (2017). 28434858 10.1016/j.cub.2017.03.047

[19] I. M. Islam, T. Erclik, Imp and Syp mediated temporal patterning of neural stem cells in the developing *Drosophila* CNS. Genetics 222, iyac103 (2022). 35881070 10.1093/genetics/iyac103 PMC9434295

[20] L.-Y. Liu, X. Long, C.-P. Yang, R. L. Miyares, K. Sugino, R. H. Singer, T. Lee, Mamo decodes hierarchical temporal gradients into terminal neuronal fate. eLife 8, e48056 (2019). 31545163 10.7554/eLife.48056 PMC6764822

[21] W. Guan, S. Bellemin, M. Bouchet, L. Venkatasubramanian, C. Guillermin, A. Laurençon, C. Kabir, A. Darnas, C. Godin, S. Urdy, R. S. Mann, J. Enriquez, Post-transcriptional regulation of transcription factor codes in immature neurons drives neuronal diversity. Cell Rep. 39, 110992 (2022). 35767953 10.1016/j.celrep.2022.110992 PMC9479746

[22] A. Hamid, H. Gattuso, A. N. Caglar, M. Pillai, T. Steele, A. Gonzalez, K. Nagel, M. H. Syed, The conserved RNA-binding protein Imp is required for the specification and function of olfactory navigation circuitry in *Drosophila*. Curr. Biol. 34, 473–488.EG (2024). 38181792 10.1016/j.cub.2023.12.020 PMC10872534

[23] U. Arain, I. M. Islam, P. Valentino, T. Erclik, Concurrent temporal patterning of neural stem cells in the fly visual system. bioRxiv 512100 [Preprint] (2022). 10.1101/2022.10.13.512100. PMC12462514 40998808

[24] J. A. Munroe, M. H. Syed, C. Q. Doe, Imp is required for timely exit from quiescence in *Drosophila* type II neuroblasts. PLOS ONE 17, e0272177 (2022). 36520944 10.1371/journal.pone.0272177 PMC9754222

[25] M. Hailstone, D. Waithe, T. J. Samuels, L. Yang, I. Costello, Y. Arava, E. Robertson, R. M. Parton, I. Davis, CytoCensus, mapping cell identity and division in tissues and organs using machine learning. eLife 9, e51085 (2020). 32423529 10.7554/eLife.51085 PMC7237217

[26] L. Landskron, V. Steinmann, F. Bonnay, T. R. Burkard, J. Steinmann, I. Reichardt, H. Harzer, A. S. Laurenson, H. Reichert, J. A. Knoblich, The asymmetrically segregating lncRNA cherub is required for transforming stem cells into malignant cells. eLife 7, e31347 (2018). 29580384 10.7554/eLife.31347 PMC5871330

[27] T. J. Samuels, A. I. Järvelin, D. Ish-Horowicz, I. Davis, Imp/IGF2BP levels modulate individual neural stem cell growth and division through *myc* mRNA stability. eLife 9, e51529 (2020). 31934860 10.7554/eLife.51529 PMC7025822

[28] M. C. Pahl, S. E. Doyle, S. E. Siegrist, E93 integrates neuroblast intrinsic state with developmental time to terminate MB neurogenesis via autophagy. Curr. Biol. 29, 750–762.e3 (2019). 30773368 10.1016/j.cub.2019.01.039 PMC6428584

[29] C. P. Yang, T. J. Samuels, Y. Huang, L. Yang, D. Ish-Horowicz, I. Davis, T. Lee, Imp and Syp RNA-binding proteins govern decommissioning of *Drosophila* neural stem cells. Development 144, 3454–3464 (2017). 28851709 10.1242/dev.149500 PMC5665480

[30] K. L. M. Boylan, S. Mische, M. Li, G. Marqués, X. Morin, W. Chia, T. S. Hays, Motility screen identifies *Drosophila* IGF-II mRNA-binding protein—zipcode-binding protein acting in oogenesis and synaptogenesis. PLOS Genet. 4, e36 (2008). 18282112 10.1371/journal.pgen.0040036 PMC2242817

[31] J. M. Halstead, Y. Q. Lin, L. Durraine, R. S. Hamilton, G. Ball, G. G. Neely, H. J. Bellen, I. Davis, Syncrip/hnRNP Q influences synaptic transmission and regulates BMP signaling at the *Drosophila* neuromuscular synapse. Biol. Open 3, 839–849 (2014). 25171887 10.1242/bio.20149027 PMC4163661

[32] H. T. Hansen, S. H. Rasmussen, S. K. Adolph, M. Plass, A. Krogh, J. Sanford, F. C. Nielsen, J. Christiansen, *Drosophila* Imp iCLIP identifies an RNA assemblage coordinating F-actin formation. Genome Biol. 16, 123 (2015). 26054396 10.1186/s13059-015-0687-0 PMC4477473

[33] C. Medioni, M. Ramialison, A. Ephrussi, F. Besse, Imp promotes axonal remodeling by regulating *profilin* mRNA during brain development. Curr. Biol. 24, 793–800 (2014). 24656828 10.1016/j.cub.2014.02.038

[34] J. Titlow, F. Robertson, A. Järvelin, D. Ish-Horowicz, C. Smith, E. Gratton, I. Davis, Syncrip/hnRNP Q is required for activity-induced Msp300/Nesprin-1 expression and new synapse formation. J. Cell Biol. 219, e201903135 (2020). 32040548 10.1083/jcb.201903135 PMC7055005

[35] T. J. Samuels, Y. Arava, A. I. Järvelin, F. Robertson, J. Y. Lee, L. Yang, C.-P. Yang, T. Lee, D. Ish-Horowicz, I. Davis, Neuronal upregulation of Prospero protein is driven by alternative mRNA polyadenylation and Syncrip-mediated mRNA stabilisation. Biol. Open 9, bio049684 (2020). 32205310 10.1242/bio.049684 PMC7225087

[36] K. R. Branham, C. Sood, S. E. Doyle, M. C. Pahl, S. E. Siegrist, Notch controls early temporal factor expression to control timing of Mushroom body neuroblast apoptosis. bioRxiv 578279 [Preprint] (2024). 10.1101/2024.01.31.578279.

[37] A. M. Rossi, C. Desplan, Extrinsic activin signaling cooperates with an intrinsic temporal program to increase mushroom body neuronal diversity. eLife 9, e58880 (2020). 32628110 10.7554/eLife.58880 PMC7365662

[38] M. Corrales, B. T. Cocanougher, A. B. Kohn, J. D. Wittenbach, X. S. Long, A. Lemire, A. Cardona, R. H. Singer, L. L. Moroz, M. Zlatic, A single-cell transcriptomic atlas of complete insect nervous systems across multiple life stages. Neural Develop. 17, 8 (2022). 10.1186/s13064-022-00164-6 PMC9404646 36002881

[39] N. Dillon, B. Cocanougher, C. Sood, X. Yuan, A. B. Kohn, L. L. Moroz, S. E. Siegrist, M. Zlatic, C. Q. Doe, Single cell RNA-seq analysis reveals temporally-regulated and quiescence-regulated gene expression in Drosophila larval neuroblasts. Neural Develop. 17, 7 (2022). 10.1186/s13064-022-00163-7 PMC9404614 36002894

[40] J. Janssens, S. Aibar, I. I. Taskiran, J. N. Ismail, A. E. Gomez, G. Aughey, K. I. Spanier, F. V. De Rop, C. B. González-Blas, M. Dionne, K. Grimes, X. J. Quan, D. Papasokrati, G. Hulselmans, S. Makhzami, M. De Waegeneer, V. Christiaens, T. Southall, S. Aerts, Decoding gene regulation in the fly brain. Nature 601, 630–636 (2022). 34987221 10.1038/s41586-021-04262-z

[41] T. A. Ravenscroft, J. Janssens, P.-T. Lee, B. Tepe, P. C. Marcogliese, S. Makhzami, T. C. Holmes, S. Aerts, H. J. Bellen, *Drosophila* voltage-gated sodium channels are only expressed in active neurons and are localized to distal axonal initial segment-like domains. J. Neurosci. 40, 7999–8024 (2020). 32928889 10.1523/JNEUROSCI.0142-20.2020 PMC7574647

[42] A. Buchbender, H. Mutter, F. X. R. Sutandy, N. Kortel, H. Hanel, A. Busch, S. Ebersberger, J. Konig, Improved library preparation with the new iCLIP2 protocol. Methods 178, 33–48 (2020). 31610236 10.1016/j.ymeth.2019.10.003

[43] S. M. McDermott, C. Meignin, J. Rappsilber, I. Davis, *Drosophila* Syncrip binds the *gurken* mRNA localisation signal and regulates localised transcripts during axis specification. Biol. Open 1, 488–497 (2012). 23213441 10.1242/bio.2012885 PMC3507208

[44] E. L. Van Nostrand, G. A. Pratt, A. A. Shishkin, C. Gelboin-Burkhart, M. Y. Fang, B. Sundararaman, S. M. Blue, T. B. Nguyen, C. Surka, K. Elkins, R. Stanton, F. Rigo, M. Guttman, G. W. Yeo, Robust transcriptome-wide discovery of RNA-binding protein binding sites with enhanced CLIP (eCLIP). Nat Methods 13, 508–514 (2016). 27018577 10.1038/nmeth.3810 PMC4887338

[45] F. C. Y. Lee, J. Ule, Advances in CLIP technologies for studies of protein-RNA interactions. Mol. Cell 69, 354–369 (2018). 29395060 10.1016/j.molcel.2018.01.005

[46] S. Sahadevan, T. Sekaran, T. Schwarzl, A pipeline for analyzing eCLIP and iCLIP data with *Htseq*-*clip* and *DEWSeq*. Methods Mol. Biol. 2404, 189–205 (2022). 34694610 10.1007/978-1-0716-1851-6_10

[47] A. E. Conway, E. L. Van Nostrand, G. A. Pratt, S. Aigner, M. L. Wilbert, B. Sundararaman, P. Freese, N. J. Lambert, S. Sathe, T. Y. Liang, A. Essex, S. Landais, C. B. Burge, D. L. Jones, G. W. Yeo, Enhanced CLIP uncovers IMP protein-RNA targets in human pluripotent stem cells important for cell adhesion and survival. Cell Rep. 15, 666–679 (2016). 27068461 10.1016/j.celrep.2016.03.052 PMC4839292

[48] M. Briese, L. Saal-Bauernschubert, C. Ji, M. Moradi, H. Ghanawi, M. Uhl, S. Appenzeller, R. Backofen, M. Sendtner, hnRNP R and its main interactor, the noncoding RNA 7SK, coregulate the axonal transcriptome of motoneurons. Proc. Natl. Acad. Sci. U.S.A. 115, E2859–E2868 (2018). 29507242 10.1073/pnas.1721670115 PMC5866599

[49] S. Khudayberdiev, M. Soutschek, I. Ammann, A. Heinze, M. B. Rust, S. Baumeister, G. Schratt, The cytoplasmic SYNCRIP mRNA interactome of mammalian neurons. RNA Biol. 18, 1252–1264 (2021). 33030396 10.1080/15476286.2020.1830553 PMC8354602

[50] R. A. Neumuller, C. Richter, A. Fischer, M. Novatchkova, K. G. Neumuller, J. A. Knoblich, Genome-wide analysis of self-renewal in *Drosophila* neural stem cells by transgenic RNAi. Cell Stem Cell 8, 580–593 (2011). 21549331 10.1016/j.stem.2011.02.022 PMC3093620

[51] J. M. Chell, A. H. Brand, Nutrition-responsive glia control exit of neural stem cells from quiescence. Cell 143, 1161–1173 (2010). 21183078 10.1016/j.cell.2010.12.007 PMC3087489

[52] S. Li, C. T. Koe, S. T. Tay, A. L. K. Tan, S. Zhang, Y. Zhang, P. Tan, W.-K. Sung, H. Wang, An intrinsic mechanism controls reactivation of neural stem cells by spindle matrix proteins. Nat. Commun. 8, 122 (2017). 28744001 10.1038/s41467-017-00172-9 PMC5526931

[53] K. Davie, J. Janssens, D. Koldere, M. De Waegeneer, U. Pech, L. Kreft, S. Aibar, S. Makhzami, V. Christiaens, C. Bravo Gonzalez-Blas, S. Poovathingal, G. Hulselmans, K. I. Spanier, T. Moerman, B. Vanspauwen, S. Geurs, T. Voet, J. Lammertyn, B. Thienpont, S. Liu, N. Konstantinides, M. Fiers, P. Verstreken, S. Aerts, A single-cell transcriptome atlas of the aging Drosophila brain. Cell 174, 982–998.e20 (2018). 29909982 10.1016/j.cell.2018.05.057 PMC6086935

[54] J. van den Ameele, A. H. Brand, Neural stem cell temporal patterning and brain tumour growth rely on oxidative phosphorylation. eLife 8, e47887 (2019). 31513013 10.7554/eLife.47887 PMC6763261

[55] S. Genovese, R. Clément, C. Gaultier, F. Besse, K. Narbonne-Reveau, F. Daian, S. Foppolo, N. M. Luis, C. Maurange, Coopted temporal patterning governs cellular hierarchy, heterogeneity and metabolism in *Drosophila* neuroblast tumors. eLife 8, e50375 (2019). 31566561 10.7554/eLife.50375 PMC6791719

[56] T. H. Nguyen, R. Vicidomini, S. D. Choudhury, T. H. Han, D. Maric, T. Brody, M. Serpe, scRNA-seq data from the larval *Drosophila* ventral cord provides a resource for studying motor systems function and development. Dev. Cell 59, 1210–1230.e9 (2024). 38569548 10.1016/j.devcel.2024.03.016 PMC11078614

[57] K. Halpern, S. Itzkovitz, Single molecule approaches for quantifying transcription and degradation rates in intact mammalian tissues. Methods 98, 134–142 (2016). 26611432 10.1016/j.ymeth.2015.11.015

[58] F. Mueller, A. Senecal, K. Tantale, H. Marie-Nelly, N. Ly, O. Collin, E. Basyuk, E. Bertrand, X. Darzacq, C. Zimmer, FISH-quant: Automatic counting of transcripts in 3D FISH images. Nat. Methods 10, 277–278 (2013). 23538861 10.1038/nmeth.2406

[59] M. K. Thompson, A. Ceccarelli, D. Ish-Horowicz, I. Davis, Dynamically regulated transcription factors are encoded by highly unstable mRNAs in *the Drosophila* larval brain. RNA 29, 1020–1032 (2023). 37041032 10.1261/rna.079552.122 PMC10275270

[60] K. Kuret, A. G. Amalietti, D. M. Jones, C. Capitanchik, J. Ule, Positional motif analysis reveals the extent of specificity of protein-RNA interactions observed by CLIP. Genome Biol. 23, 191 (2022). 36085079 10.1186/s13059-022-02755-2 PMC9461102

[61] A. Siepel, G. Bejerano, J. S. Pedersen, A. S. Hinrichs, M. Hou, K. Rosenbloom, H. Clawson, J. Spieth, L. W. Hillier, S. Richards, G. M. Weinstock, R. K. Wilson, R. A. Gibbs, W. J. Kent, W. Miller, D. Haussler, Evolutionarily conserved elements in vertebrate, insect, worm, and yeast genomes. Genome Res. 15, 1034–1050 (2005). 16024819 10.1101/gr.3715005 PMC1182216

[62] C. Weinreb, A. J. Riesselman, J. B. Ingraham, T. Gross, C. Sander, D. S. Marks, 3D RNA and functional interactions from evolutionary couplings. Cell 165, 963–975 (2016). 27087444 10.1016/j.cell.2016.03.030 PMC5024353

[63] C. Alfonso-Gonzalez, I. Legnini, S. Holec, L. Arrigoni, H. C. Ozbulut, F. Mateos, D. Koppstein, A. Rybak-Wolf, U. Bönisch, N. Rajewsky, V. Hilgers, Sites of transcription initiation drive mRNA isoform selection. Cell 186, 2438–2455.e22 (2023). 37178687 10.1016/j.cell.2023.04.012 PMC10228280

[64] R. Brandman, Y. Brandman, V. S. Pande, Sequence coevolution between RNA and protein characterized by mutual information between residue triplets. PLOS ONE 7, e30022 (2012). 22279560 10.1371/journal.pone.0030022 PMC3261191

[65] L. C. Martin, G. B. Gloor, S. D. Dunn, L. M. Wahl, Using information theory to search for co-evolving residues in proteins. Bioinformatics 21, 4116–4124 (2005). 16159918 10.1093/bioinformatics/bti671

[66] G. S. Marques, J. Teles-Reis, N. Konstantinides, P. H. Brito, C. C. F. Homem, Asynchronous transcription and translation of neurotransmitter-related genes characterize the initial stages of neuronal maturation in *Drosophila*. PLOS Biol. 21, e3002115 (2023). 37205703 10.1371/journal.pbio.3002115 PMC10234549

[67] O. A. Bayraktar, C. Q. Doe, Combinatorial temporal patterning in progenitors expands neural diversity. Nature 498, 449–455 (2013). 23783519 10.1038/nature12266 PMC3941985

[68] T. Erclik, X. Li, M. Courgeon, C. Bertet, Z. Chen, R. Baumert, J. Ng, C. Koo, U. Arain, R. Behnia, A. del Valle Rodriguez, L. Senderowicz, N. Negre, K. P. White, C. Desplan, Integration of temporal and spatial patterning generates neural diversity. Nature 541, 365–370 (2017). 28077877 10.1038/nature20794 PMC5489111

[69] R. Rahman, W. Xu, H. Jin, M. Rosbash, Identification of RNA-binding protein targets with HyperTRIBE. Nat. Protoc. 13, 1829–1849 (2018). 30013039 10.1038/s41596-018-0020-y PMC6349038

[70] Y.-J. Lee, C.-P. Yang, R. L. Miyares, Y.-F. Huang, Y. He, Q. Ren, H.-M. Chen, T. Kawase, M. Ito, H. Otsuna, K. Sugino, Y. Aso, K. Ito, T. Lee, Conservation and divergence of related neuronal lineages in the *Drosophila* central brain. eLife 9, e53518 (2020). 32255422 10.7554/eLife.53518 PMC7173964

[71] C. M. Di Liegro, G. Schiera, I. Di Liegro, Regulation of mRNA transport, localization and translation in the nervous system of mammals (Review). Int. J. Mol. Med. 33, 747–762 (2014). 24452120 10.3892/ijmm.2014.1629 PMC3976132

[72] C. Dillard, K. Narbonne-Reveau, S. Foppolo, E. Lanet, C. Maurange, Two distinct mechanisms silence *chinmo* in *Drosophila* neuroblasts and neuroepithelial cells to limit their self-renewal. Development 145, dev154534 (2018). 29361557 10.1242/dev.154534

[73] E. L. Van Nostrand, G. A. Pratt, B. A. Yee, E. C. Wheeler, S. M. Blue, J. Mueller, S. S. Park, K. E. Garcia, C. Gelboin-Burkhart, T. B. Nguyen, I. Rabano, R. Stanton, B. Sundararaman, R. Wang, X.-D. Fu, B. R. Graveley, G. W. Yeo, Principles of RNA processing from analysis of enhanced CLIP maps for 150 RNA binding proteins. Genome Biol. 21, 90 (2020). 32252787 10.1186/s13059-020-01982-9 PMC7137325

[74] E. Dassi, Handshakes and fights: The regulatory interplay of RNA-binding proteins. Front. Mol. Biosci. 4, 67 (2017). 29034245 10.3389/fmolb.2017.00067 PMC5626838

[75] C. Tiedje, N. Ronkina, M. Tehrani, S. Dhamija, K. Laass, H. Holtmann, A. Kotlyarov, M. Gaestel, The p38/MK2-driven exchange between tristetraprolin and HuR regulates AU–rich element–dependent translation. PLOS Genet. 8, e1002977 (2012). 23028373 10.1371/journal.pgen.1002977 PMC3459988

[76] V. Iadevaia, A. P. Gerber, Combinatorial control of mRNA fates by RNA-binding proteins and non-coding RNAs. Biomolecules 5, 2207–2222 (2015). 26404389 10.3390/biom5042207 PMC4693235

[77] N. Degrauwe, M.-L. Suvà, M. Janiszewska, N. Riggi, I. Stamenkovic, IMPs: An RNA-binding protein family that provides a link between stem cell maintenance in normal development and cancer. Genes Dev. 30, 2459–2474 (2016). 27940961 10.1101/gad.287540.116 PMC5159662

[78] Y. V. Svitkin, A. Yanagiya, A. E. Karetnikov, T. Alain, M. R. Fabian, A. Khoutorsky, S. Perreault, I. Topisirovic, N. Sonenberg, Control of translation and miRNA-dependent repression by a novel poly(A) binding protein, hnRNP-Q. PLOS Biol. 11, e1001564 (2013). 23700384 10.1371/journal.pbio.1001564 PMC3660254

[79] D. Weidensdorfer, N. Stöhr, A. Baude, M. Lederer, M. Köhn, A. Schierhorn, S. Buchmeier, E. Wahle, S. Hüttelmaier, Control of c-myc mRNA stability by IGF2BP1-associated cytoplasmic RNPs. RNA 15, 104–115 (2009). 19029303 10.1261/rna.1175909 PMC2612774

[80] H.-H. Chen, H.-I. Yu, W.-C. Chiang, Y.-D. Lin, B.-C. Shia, W.-Y. Tarn, hnRNP Q regulates Cdc42-mediated neuronal morphogenesis. Mol. Cell. Biol. 32, 2224–2238 (2012). 22493061 10.1128/MCB.06550-11 PMC3372263

[81] H. Mori, S.-i. Sakakibara, T. Imai, Y. Nakamura, T. Iijima, A. Suzuki, Y. Yuasa, M. Takeda, H. Okano, Expression of mouse *igf2* mRNA-binding protein 3 and its implications for the developing central nervous system. J. Neurosci. Res. 64, 132–143 (2001). 11288142 10.1002/jnr.1060

[82] J. Nishino, S. Kim, Y. Zhu, H. Zhu, S. J. Morrison, A network of heterochronic genes including *Imp1* regulates temporal changes in stem cell properties. eLife 2, e00924 (2013). 24192035 10.7554/eLife.00924 PMC3817382

[83] V. S. Caviness Jr., R. S. Nowakowski, P. G. Bhide, Neocortical neurogenesis: Morphogenetic gradients and beyond. Trends Neurosci. 32, 443–450 (2009). 19635637 10.1016/j.tins.2009.05.003 PMC2725216

[84] L. Telley, G. Agirman, J. Prados, N. Amberg, S. Fievre, P. Oberst, G. Bartolini, I. Vitali, C. Cadilhac, S. Hippenmeyer, L. Nguyen, A. Dayer, D. Jabaudon, Temporal patterning of apical progenitors and their daughter neurons in the developing neocortex. Science 364, eaav2522 (2019). 31073041 10.1126/science.aav2522

[85] S. K. Zahr, G. Yang, H. Kazan, M. J. Borrett, S. A. Yuzwa, A. Voronova, D. R. Kaplan, F. D. Miller, A translational repression complex in developing mammalian neural stem cells that regulates neuronal specification. Neuron 97, 520–537.e6 (2018). 29395907 10.1016/j.neuron.2017.12.045

[86] A. Busch, M. Bruggemann, S. Ebersberger, K. Zarnack, iCLIP data analysis: A complete pipeline from sequencing reads to RBP binding sites. Methods 178, 49–62 (2020). 31751605 10.1016/j.ymeth.2019.11.008

[87] G. W. Yeo, N. G. Coufal, T. Y. Liang, G. E. Peng, X.-D. Fu, F. H. Gage, An RNA code for the FOX2 splicing regulator revealed by mapping RNA-protein interactions in stem cells. Nat. Struct. Mol. Biol. 16, 130–137 (2009). 19136955 10.1038/nsmb.1545 PMC2735254

[88] M. I. Love, W. Huber, S. Anders, Moderated estimation of fold change and dispersion for RNA-seq data with DESeq2. Genome Biol 15, 550 (2014). 25516281 10.1186/s13059-014-0550-8 PMC4302049

[89] T. Schwarzl, S. Sahadevan, B. Lang, M. Miladi, R. Backofen, W. Huber, M. W. Hentze, G. G. Tartaglia, Improved discovery of RNA-binding protein binding sites in eCLIP data using DEWSeq. Nucleic Acids Res. 52, e1 (2024). 37962298 10.1093/nar/gkad998 PMC10783507

[90] I. Gaspar, F. Wippich, A. Ephrussi, Enzymatic production of single-molecule FISH and RNA capture probes. RNA 23, 1582–1591 (2017). 28698239 10.1261/rna.061184.117 PMC5602115

