# Supplementary material for: Imp/IGF2BP and Syp/SYNCRIP temporal RNA interactomes uncover combinatorial networks of regulators of Drosophila brain development
Source: Sci Adv. 2025 Feb 7;11(6):eadr6682. doi: 10.1126/sciadv.adr6682 (PMC11804933; doi:10.1126/sciadv.adr6682)
Supplement: Supplementary file 1 — Figs. S1 to S7 Legends for data S1 to S5 [file sciadv.adr6682_sm.pdf]

Science Advances
AAAS

Supplementary Materials for

Imp/IGF2BP and Syp/SYNCRIP temporal RNA interactomes uncover combinatorial networks of regulators of *Drosophila* brain development

Jeffrey Y. Lee *et al.*

Corresponding author: Jeffrey Y. Lee, Jeff.Lee@glasgow.ac.uk; Ilan Davis, Ilan.Davis@glasgow.ac.uk

Sci. Adv. **11**, eadr6682 (2025)
DOI: 10.1126/sciadv.adr6682

The PDF file includes:

Figs. S1 to S7
Legends for data S1 to S5

Other Supplementary Material for this manuscript includes the following:

Data S1 to S5

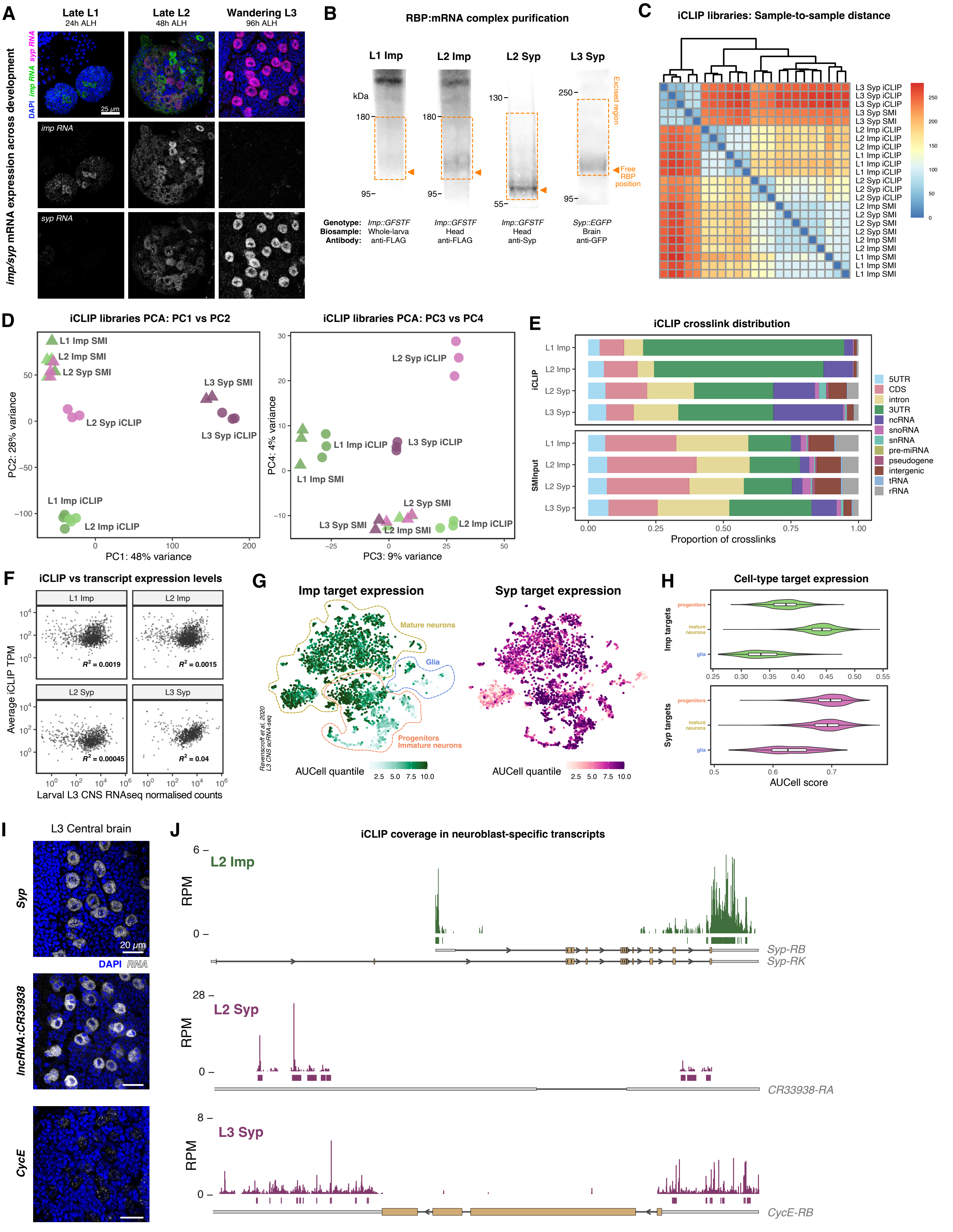

A
Late L1
24h ALH
Late L2
48h ALH
Wandering L3
96h ALH
imp/syp mRNA expression across development
DAPI imp RNA syp RNA
25 μm
imp RNA
syp RNA
B
RBP:mRNA complex purification
L1 Imp
L2 Imp
L2 Syp
L3 Syp
kDa
180
95
130
55
250
Excised region
Free RBP position
Genotype: Imp::GFSTF Imp::GFSTF Imp::GFSTF Syp::EGFP
Biosample: Whole-larva Head Head Brain
Antibody: anti-FLAG anti-FLAG anti-Syp anti-GFP
C
iCLIP libraries: Sample-to-sample distance
D
iCLIP libraries PCA: PC1 vs PC2
iCLIP libraries PCA: PC3 vs PC4
PC1: 48% variance
PC2: 28% variance
PC3: 9% variance
PC4: 4% variance
E
iCLIP crosslink distribution
Proportion of crosslinks
5UTR
CDS
intron
3UTR
ncRNA
snoRNA
snRNA
pre-miRNA
pseudogene
intergenic
tRNA
rRNA
F
iCLIP vs transcript expression levels
Average iCLIP TPM
Larval L3 CNS RNAseq normalised counts
G
Imp target expression
Syp target expression
Mature neurons
Glia
Progenitors
Immature neurons
AUCell quantile
Ravenscroft et al, 2020
L3 CNS scRNA-seq
H
Cell-type target expression
Imp targets
Syp targets
progenitors
mature neurons
glia
AUCell score
I
L3 Central brain
Syp
lncRNA:CR33938
CycE
20 μm
DAPI RNA
J
iCLIP coverage in neuroblast-specific transcripts
L2 Imp
L2 Syp
L3 Syp
RPM
Syp-RB
Syp-RK
CR33938-RA
CycE-RB

Figure S1

Fig. S1. Identification of *in vivo* RNA targets of Imp and Syp in larval brains, related to Fig. 1.

(**A**) Temporal RNA expression patterns of *imp* and *syp* in the *Drosophila* larval nervous system, visualised by single-molecule fluorescence *in situ* hybridisation (smFISH).

(**B**) Infrared blot of RNA-binding protein (RBP):RNA complexes purified via immunoprecipitation. Orange regions indicate the excised molecular weight range used for iCLIP library preparation, with arrows marking the expected molecular weights of unbound Imp or Syp.

(**C**) Heatmap of hierarchical clustering for iCLIP and size-matched input (SMInput) libraries, showing sample-to-sample distances calculated from transformed count matrices.

(**D**) Principal component analysis (PCA) of iCLIP libraries based on normalised crosslink counts per gene, with PC1 vs. PC2 or PC3 vs. PC4 shown in left and right panels. Each iCLIP library is colour-coded, while SMInput libraries are shown with a different shape.

(**E**) Relative proportion of crosslinks in iCLIP and SMInput libraries by transcript biotypes. Protein coding genes are further sub-divided into 5'UTR, CDS, 3'UTR, and intron categories.

(**F**) Correlation of iCLIP reads (normalised to transcripts per million, TPM) versus transcript abundance obtained from Wildtype L3 brains *(35)* per gene. Each point represents the average of three replicates, with R^2 values shown for each iCLIP library.

(**G**) Area Under Curve Cell (AUCell) scores of Imp and Syp targets across the L3 brain single-cell RNA-seq atlas *(41)*. Broad progenitors/immature neurons, mature neurons and glial cell types identified from the original publication are indicated.

(**H**) Comparison of AUCell scores for Imp and Syp targets across progenitors, mature neurons or glia cell-types.

(**I**) RNA expression patterns of neuroblast (NB) specific transcripts, *Syp*, *lncRNA:CR33938* and *CycE*, visualised by smFISH in the L3 brain.

(**J**) Imp and Syp iCLIP coverage in NB-specific transcripts.

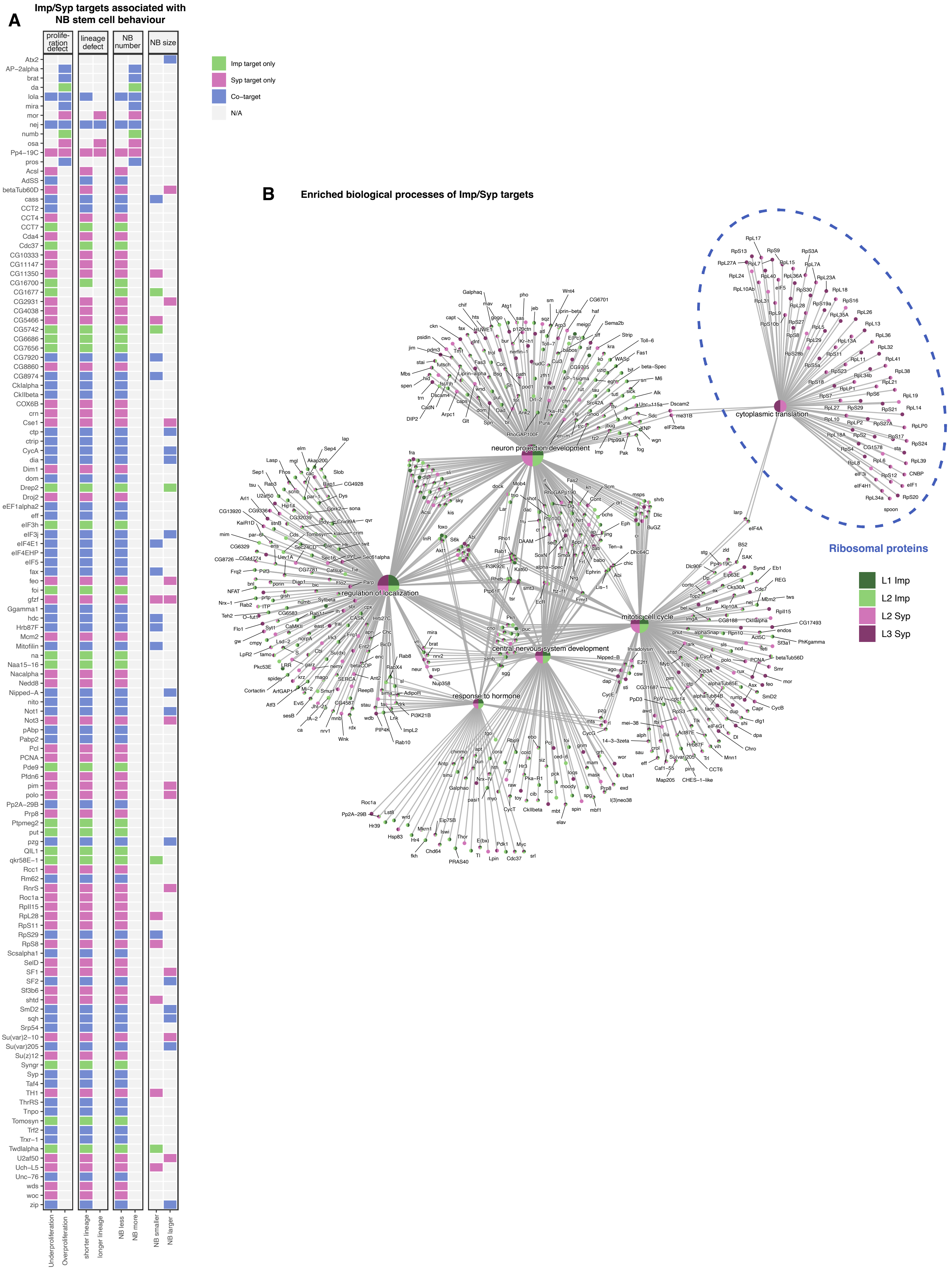
A
Imp/Syp targets associated with NB stem cell behaviour
proliferation defect
lineage defect
NB number
NB size
Underproliferation
Overproliferation
shorter lineage
longer lineage
NB less
NB more
NB smaller
NB larger
Imp target only
Syp target only
Co−target
N/A
Atx2
AP−2alpha
brat
da
lola
mira
mor
nej
numb
osa
Pp4−19C
pros
Acsl
AdSS
betaTub60D
cass
CCT2
CCT4
CCT7
Cda4
Cdc37
CG10333
CG11147
CG11350
CG16700
CG1677
CG2931
CG4038
CG5466
CG5742
CG6686
CG7656
CG7920
CG8860
CG8974
CkIalpha
CkIIbeta
COX6B
crn
Cse1
ctp
ctrip
CycA
dia
Dim1
dom
Drep2
Droj2
eEF1alpha2
eff
eIF3h
eIF3j
eIF4E1
eIF4EHP
eIF5
fax
feo
foi
gfzf
Ggamma1
hdc
Hrb87F
Mcm2
Mitofilin
na
Naa15−16
Nacalpha
Nedd8
Nipped−A
nito
Not1
Not3
pAbp
Pabp2
Pcl
PCNA
Pde9
Pfdn6
pim
polo
Pp2A−29B
Prp8
Ptpmeg2
put
pzg
QIL1
qkr58E−1
Rcc1
Rm62
RnrS
Roc1a
RpII15
RpL28
RpS11
RpS29
RpS8
Scsalpha1
SelD
SF1
SF2
Sf3b6
shtd
SmD2
sqh
Srp54
Su(var)2−10
Su(var)205
Su(z)12
Syngr
Syp
Taf4
TH1
ThrRS
Tnpo
Tomosyn
Trf2
Trxr−1
Twdlapha
U2af50
Uch−L5
Unc−76
wds
woc
zip
B
Enriched biological processes of Imp/Syp targets
cytoplasmic translation
neuron projection development
regulation of localization
mitotic cell cycle
central nervous system development
response to hormone
Ribosomal proteins
L1 Imp
L2 Imp
L2 Syp
L3 Syp

Figure S2

Fig. S2. The analysis of Imp and Syp target functions in the neuroblast lineage, related to Fig. 2.

(**A**) Imp and Syp targets involved in regulating NB size, NB number, lineage length, or neural progenitor proliferation, identified via genome-wide RNAi screen *(50)*. Only the genes that are Imp or Syp targets are shown.

(**B**) Network plot of top enriched GO biological process terms and associated genes. The shades of green and magenta colours represent Imp or Syp iCLIP target status. A group of transcripts encoding ribosomal proteins is indicated in blue, which exclusively interacts with Syp.

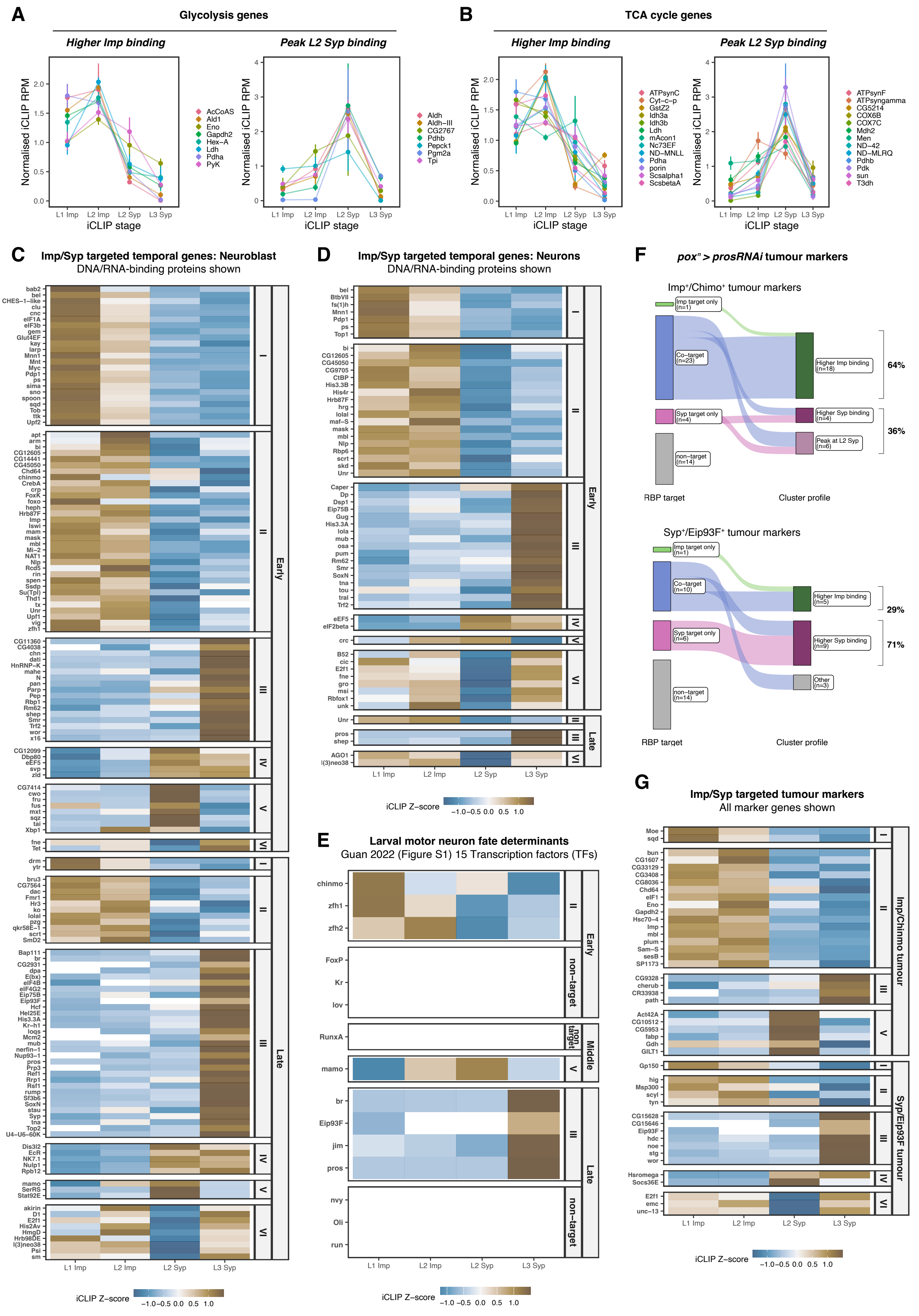

A
Glycolysis genes
Higher Imp binding
Peak L2 Syp binding
Normalised iCLIP RPM
iCLIP stage
L1 Imp
L2 Imp
L2 Syp
L3 Syp
AcCoAS
Ald1
Eno
Gapdh2
Hex−A
Ldh
Pdha
PyK
Aldh
Aldh−III
CG2767
Pdhb
Pepck1
Pgm2a
Tpi
B
TCA cycle genes
ATPsynC
Cyt−c−p
GstZ2
Idh3a
Idh3b
Ldh
mAcon1
Nc73EF
ND−MNLL
Pdha
porin
Scsalpha1
ScsbetaA
ATPsynF
ATPsyngamma
CG5214
COX6B
COX7C
Mdh2
Men
ND−42
ND−MLRQ
Pdhb
Pdk
sun
T3dh
C
Imp/Syp targeted temporal genes: Neuroblast
DNA/RNA-binding proteins shown
Early
Late
iCLIP Z−score
D
Imp/Syp targeted temporal genes: Neurons
DNA/RNA-binding proteins shown
E
Larval motor neuron fate determinants
Guan 2022 (Figure S1) 15 Transcription factors (TFs)
chinmo
zfh1
zfh2
FoxP
Kr
lov
RunxA
mamo
br
Eip93F
jim
pros
nvy
Oli
run
non−target
Middle
F
pox^n > prosRNAi tumour markers
Imp+/Chimo+ tumour markers
Imp target only (n=1)
Co−target (n=23)
Syp target only (n=4)
non−target (n=14)
Higher Imp binding (n=18)
Higher Syp binding (n=4)
Peak at L2 Syp (n=6)
64%
36%
RBP target
Cluster profile
Syp+/Eip93F+ tumour markers
Imp target only (n=1)
Co−target (n=10)
Syp target only (n=6)
non−target (n=14)
Higher Imp binding (n=5)
Higher Syp binding (n=9)
Other (n=3)
29%
71%
G
Imp/Syp targeted tumour markers
All marker genes shown
Imp/Chimo tumour
Syp/Eip93F tumour

Figure S3

Fig. S3. Temporally expressed transcripts interact dynamically with Imp and Syp, related to Fig. 3.
(**A-B**) Transcripts encoding energy metabolism regulators involved in (**A**) glycolysis or (**B**) the TCA cycle, showing 'High Imp binding' or 'Peak L2 Syp binding' interaction patterns.
(**C-D**) Heatmaps of scaled iCLIP scores for temporally regulated factors in (**C**) NBs or (**D**) immature/mature neurons grouped by *k*-means cluster. Only transcripts encoding DNA or RNA-binding proteins are shown.
(**E**) Heatmap of scaled iCLIP scores for larval motor neuron fate determinant transcription factors (TFs) (*21*). "Early", "Middle" and "Late" classifications are based on Fig. S1 from Guan *et al.*, 2022 (*21*), with all 15 TFs in the combinatorial TF code shown.
(**F**) Sankey plot showing hierarchical tumour markers (Imp^{+}/$Chinmo^{+}$ versus Syp^{+}/$Eip93F^{+}$) that dynamically interact with Imp and Syp. Tumour markers were identified from pox^{n} > *pros* RNAi scRNA-seq dataset (*55*).
(**G**) Heatmaps of scaled iCLIP scores for Imp^{+}/$Chinmo^{+}$ and Syp^{+}/$Eip93F^{+}$ tumour markers, grouped by *k*-means cluster membership.

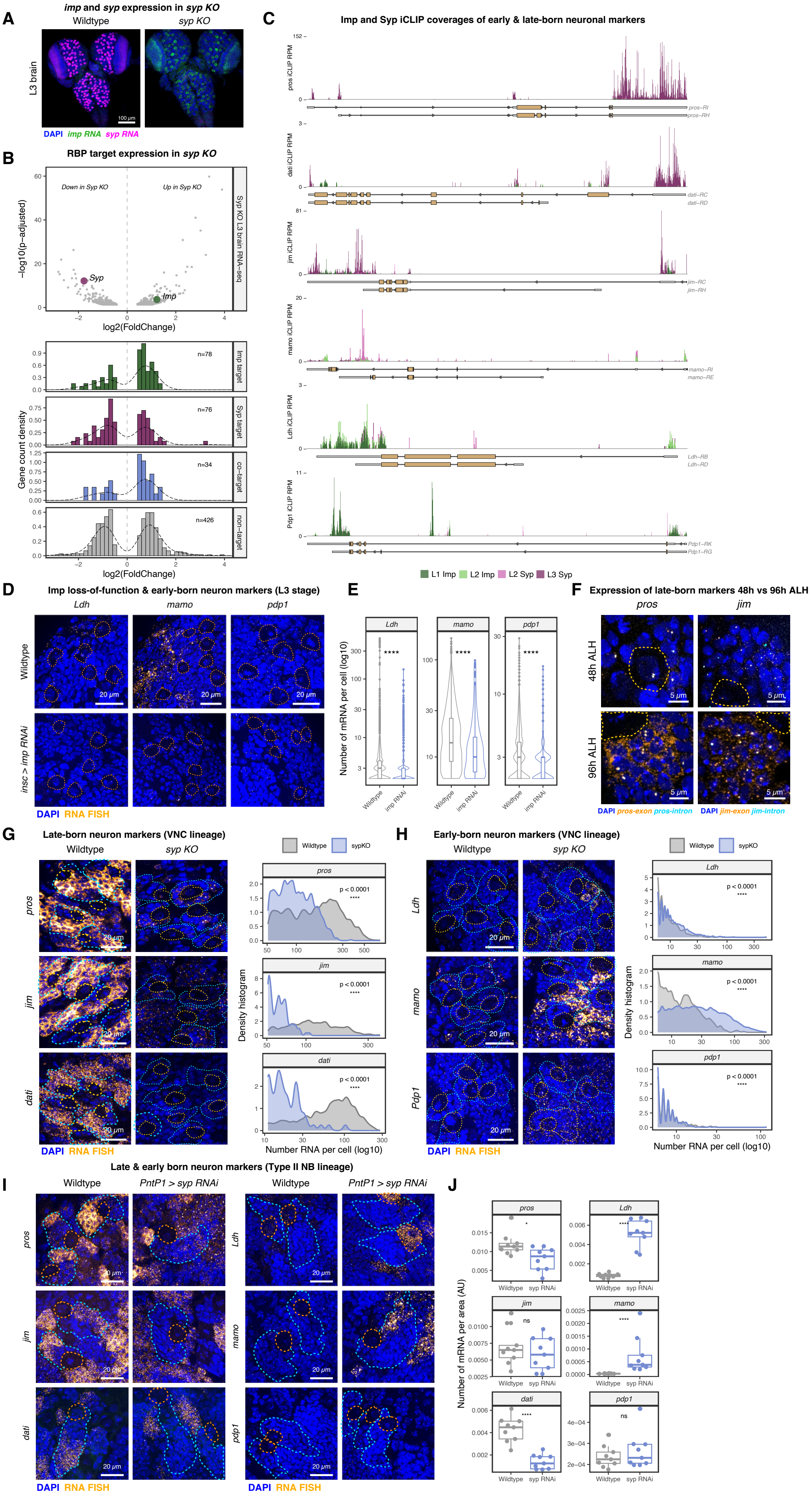
A
imp and syp expression in syp KO
Wildtype
syp KO
L3 brain
100 μm
DAPI imp RNA syp RNA
B
RBP target expression in syp KO
Down in Syp KO
Up in Syp KO
Syp
Imp
Syp KO L3 brain RNA-seq
−log10(p−adjusted)
log2(FoldChange)
n=78
Imp target
n=76
Syp target
n=34
co−target
n=426
non−target
Gene count density
log2(FoldChange)
C
Imp and Syp iCLIP coverages of early & late-born neuronal markers
pros iCLIP RPM
pros−RI
pros−RH
dati iCLIP RPM
dati−RC
dati−RD
jim iCLIP RPM
jim−RC
jim−RH
mamo iCLIP RPM
mamo−RI
mamo−RE
Ldh iCLIP RPM
Ldh−RB
Ldh−RD
Pdp1 iCLIP RPM
Pdp1−RK
Pdp1−RG
L1 Imp
L2 Imp
L2 Syp
L3 Syp
D
Imp loss-of-function & early-born neuron markers (L3 stage)
Ldh
mamo
pdp1
Wildtype
insc > imp RNAi
20 μm
DAPI RNA FISH
E
Ldh
mamo
pdp1
Number of mRNA per cell (log10)
Wildtype
imp RNAi
F
Expression of late-born markers 48h vs 96h ALH
pros
jim
48h ALH
96h ALH
5 μm
DAPI pros-exon pros-intron
DAPI jim-exon jim-intron
G
Late-born neuron markers (VNC lineage)
Wildtype
syp KO
pros
jim
dati
20 μm
DAPI RNA FISH
Wildtype
sypKO
p < 0.0001
Density histogram
Number RNA per cell (log10)
H
Early-born neuron markers (VNC lineage)
Wildtype
syp KO
Ldh
mamo
Pdp1
20 μm
DAPI RNA FISH
Wildtype
sypKO
p < 0.0001
Density histogram
Number RNA per cell (log10)
Late & early born neuron markers (Type II NB lineage)
I
Wildtype
PntP1 > syp RNAi
pros
jim
dati
Ldh
mamo
pdp1
20 μm
DAPI RNA FISH
J
pros
Ldh
jim
mamo
dati
pdp1
ns
Number of mRNA per area (AU)
Wildtype
syp RNAi

Figure S4

Fig. S4. Imp and Syp bind and regulate temporally expressed transcripts, related to Fig. 4.
(**A**) *imp* and *syp* mRNA expression in Wildtype and *syp KO* L3 brains.
(**B**) Differential expression of Imp and Syp targets in L3 *syp KO* brains. Histograms show log2 fold-change in transcript abundance for Imp, Syp or co-targets.
(**C**) iCLIP coverage of Imp and Syp across early and late-born neuronal markers.
(**D**) smFISH visualisation of early-born neuronal marker transcripts in Wildtype and *insc > imp RNAi* L3 brains, focusing on central brain Type I NB lineages. NBs are outlined with dashed lines.
(**E**) Quantification of early-born neuronal marker mRNAs in Wildtype and *insc > imp RNAi* L3 brains. smFISH data were quantified as the number of mRNAs per cell (log-scale), represented as violin plots. (n=3). ****p<0.0001.
(**F**) Transcription and mRNA accumulation of *pros* and *jim* in central brain Type I NBs at 48h ALH versus 96h ALH. Intronic smFISH probes indicate nuclear transcription sites, while exonic probes show cytoplasmic mRNA accumulation. (n=2).
(**G**) Altered mRNA expression of late-born (*pros, jim, dati*) neuronal markers in Wildtype and *syp KO* L3 ventral nerve cord (VNC) NB lineages. smFISH images were quantified as the number of mRNAs per cell (log-scale), shown as density histogram per genotype. All cells in the field of view were analysed. VNC NBs and progenies are outlined with dashed lines. Kolmogorow-Smirnov test of differential distribution. (n=3). ****p<0.0001.
(**H**) Altered mRNA expression of early-born (*Ldh, mamo, Pdp1*) neuronal markers in Wildtype and *syp KO* L3 VNC NB lineages. (n=3). ****p<0.0001.
(**I**) Altered expression of late/early-born neuronal markers in *syp*-RNAi L3 Type II NB lineages. *PntP1-GAL4* driving RNAi in Type II NB lineages. Type II NBs and their progenies are outlined with dashed lines.
(**J**) Quantification of mRNA expression for late/early-born neuronal markers in Wildtype and *PntP1>syp-RNAi* Type II NB lineages. smFISH data were quantified as mRNA density per *PntP1*-positive area. (n=3). *p<0.05, ****p<0.0001.

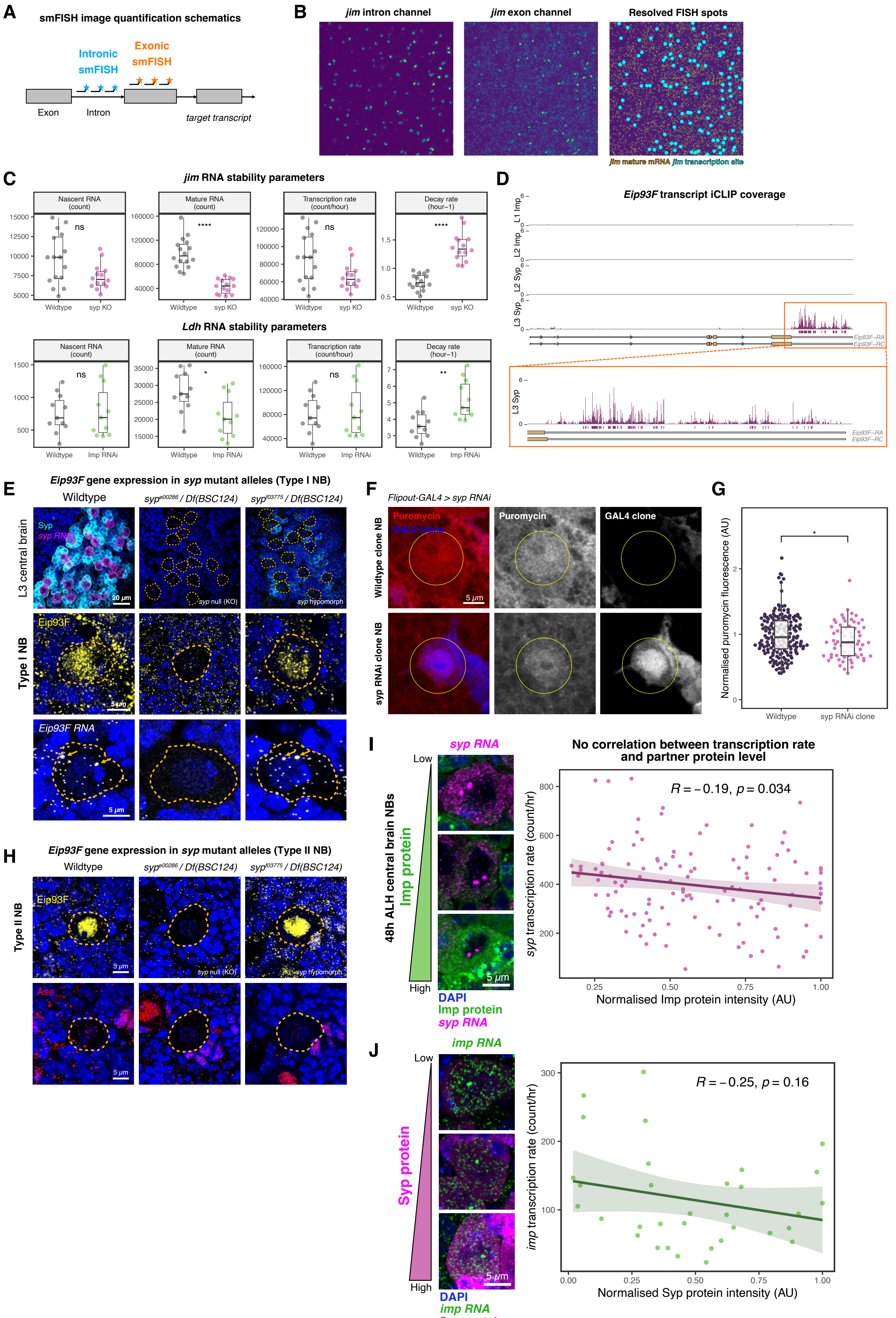

A
smFISH image quantification schematics
Intronic smFISH
Exonic smFISH
Exon
Intron
target transcript
B
jim intron channel
jim exon channel
Resolved FISH spots
jim mature mRNA jim transcription site
C
jim RNA stability parameters
Nascent RNA (count)
Mature RNA (count)
Transcription rate (count/hour)
Decay rate (hour−1)
Wildtype
syp KO
Ldh RNA stability parameters
Imp RNAi
D
Eip93F transcript iCLIP coverage
L1 Imp
L2 Imp
L2 Syp
L3 Syp
Eip93F-RA
Eip93F-RC
E
Eip93F gene expression in syp mutant alleles (Type I NB)
Wildtype
syp^e00286 / Df(BSC124)
syp^f03775 / Df(BSC124)
L3 central brain
Type I NB
Syp
syp RNA
Eip93F
Eip93F RNA
syp null (KO)
syp hypomorph
F
Flipout-GAL4 > syp RNAi
Puromycin
GAL4 clone
Wildtype clone NB
syp RNAi clone NB
G
Normalised puromycin fluorescence (AU)
Wildtype
syp RNAi clone
H
Eip93F gene expression in syp mutant alleles (Type II NB)
Type II NB
Ase
I
syp RNA
No correlation between transcription rate and partner protein level
48h ALH central brain NBs
Imp protein
Low
High
R = −0.19, p = 0.034
syp transcription rate (count/hr)
Normalised Imp protein intensity (AU)
DAPI
Imp protein
syp RNA
J
imp RNA
Syp protein
R = −0.25, p = 0.16
imp transcription rate (count/hr)
Normalised Syp protein intensity (AU)
DAPI
imp RNA
Syp protein

Figure S5

Fig. S5. Imp and Syp post-transcriptionally regulate downstream targets, related to Fig. 5.
(**A**) smFISH strategy to calculate steady-state RNA stability using exon and intron probes.
(**B**) Example images of *jim* mRNA intron and exon smFISH channels in the larval central brain. Both channels were analysed to count mature and nascent transcripts using BigFISH (see Methods). Viridis look-up table is used.
(**C**) Quantification of *jim* (n=3) and *Ldh* (n=2) RNA stability parameters. *p<0.05, **p<0.01, ****p<0.0001.
(**D**) iCLIP coverages of Imp and Syp on *Eip93F* mRNA. Note there are no significant Imp and L2 Syp binding sites on *Eip93F*.
(**E**) Expression pattern of *Eip93F* RNA and protein in Wildtype, *syp KO* (*syp*^{e00286}*/Df(BSC124)*), and *syp hypomorph* (*syp*^{f03775}*/Df(BSC124)*) central brain Type I NBs. Yellow arrows indicate nuclear *Eip93F* transcription sites, which is absent in *syp KO* NBs. (n=3).
(**F**) Puromycin incorporation in Wildtype versus flip-out *syp* RNAi NB clones in the L3 brain, visualised using anti-puromycin immunofluorescence.
(**G**) Quantification of puromycin incorporation in Wildtype versus flip-out *syp* RNAi NB clones (n=3). *p<0.05.
(**H**) Eip93F protein expression pattern in Wildtype, *syp KO*, and *syp hypomorph* central brain Type II NBs, identified as NBs lacking Asense (Ase) immunofluorescence. (n=3).
(**I**) Correlation between Imp protein and *syp* transcription rate in individual NBs at 48h ALH. Central brain Type I NBs of Imp::GFSTF genotype with smFISH against *syp*. Pearson correlation test. (n=3).
(**J**) Correlation between Syp protein and *imp* transcription rate individual NBs at 48h ALH. Central brain Type I NBs of Syp::GFP genotype with smFISH against *imp*. Pearson correlation test. (n=2).

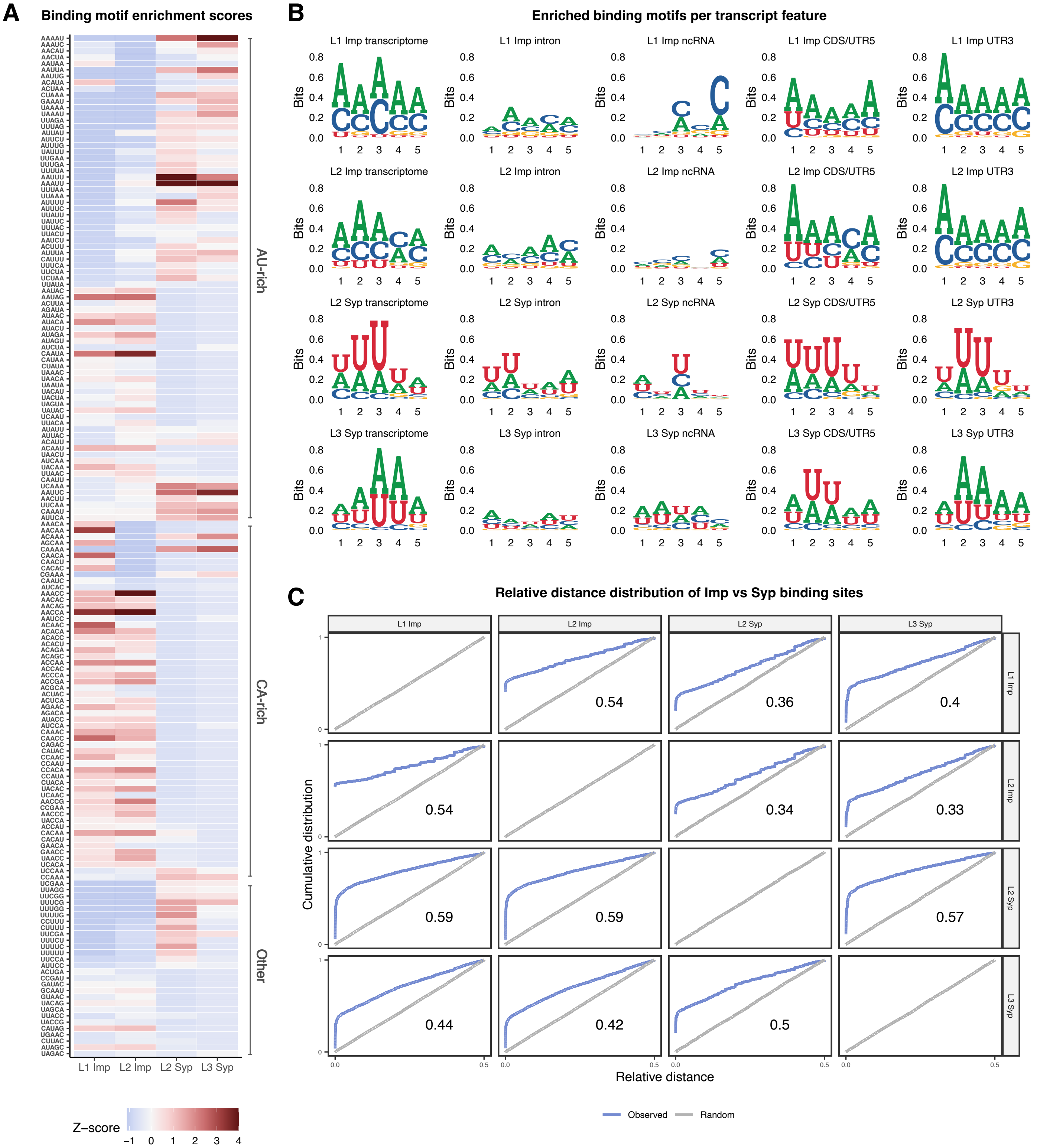
A
Binding motif enrichment scores
AU-rich
CA-rich
Other
L1 Imp
L2 Imp
L2 Syp
L3 Syp
Z-score
−1 0 1 2 3 4
B
Enriched binding motifs per transcript feature
L1 Imp transcriptome
L1 Imp intron
L1 Imp ncRNA
L1 Imp CDS/UTR5
L1 Imp UTR3
L2 Imp transcriptome
L2 Imp intron
L2 Imp ncRNA
L2 Imp CDS/UTR5
L2 Imp UTR3
L2 Syp transcriptome
L2 Syp intron
L2 Syp ncRNA
L2 Syp CDS/UTR5
L2 Syp UTR3
L3 Syp transcriptome
L3 Syp intron
L3 Syp ncRNA
L3 Syp CDS/UTR5
L3 Syp UTR3
Bits
C
Relative distance distribution of Imp vs Syp binding sites
L1 Imp
L2 Imp
L2 Syp
L3 Syp
0.54
0.36
0.4
0.54
0.34
0.33
0.59
0.59
0.57
0.44
0.42
0.5
Cumulative distribution
Relative distance
Observed
Random

Figure S6

Fig. S6. Imp and Syp binding sites poorly overlap, except in temporally expressed transcripts, related to Fig. 6.

(**A**) Heatmap of RNA sequence motif enrichment scores (scaled) for each iCLIP library. Only the top enriched 5-mers are shown. The plot is subdivided into AU-rich, CA-rich, and other enriched sequence motifs.

(**B**) Enriched RNA binding sequence motifs of Imp and Syp across different developmental stages and transcript features.

(**C**) Relative distance metrics between Imp and Syp iCLIP binding sites. Cumulative plots that compare observed (empirical) relative distances versus randomly simulated binding sites are shown. Transcriptome-wide relative distance metrics were calculated using the *genometricorr* R package. The correlation index can range from -1 to +1 where the value of -1 indicates perfectly even spacing between binding sites, while values closer to +1 indicate closer proximity between genomic intervals.

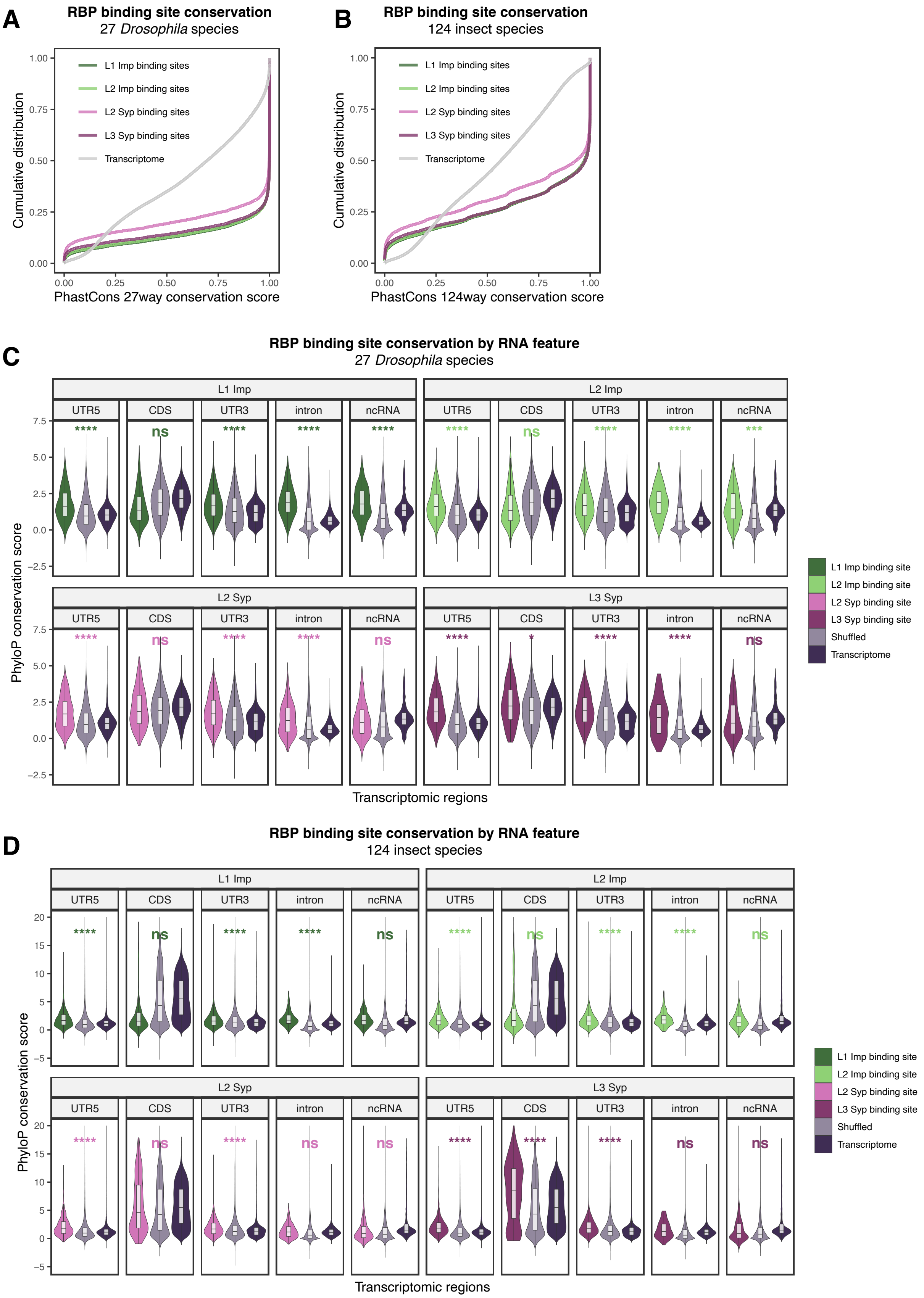
A
RBP binding site conservation
27 Drosophila species
L1 Imp binding sites
L2 Imp binding sites
L2 Syp binding sites
L3 Syp binding sites
Transcriptome
Cumulative distribution
PhastCons 27way conservation score
B
RBP binding site conservation
124 insect species
Cumulative distribution
PhastCons 124way conservation score
C
RBP binding site conservation by RNA feature
27 Drosophila species
L1 Imp
L2 Imp
L2 Syp
L3 Syp
UTR5
CDS
UTR3
intron
ncRNA
PhyloP conservation score
Transcriptomic regions
L1 Imp binding site
L2 Imp binding site
L2 Syp binding site
L3 Syp binding site
Shuffled
Transcriptome
D
RBP binding site conservation by RNA feature
124 insect species
PhyloP conservation score
Transcriptomic regions

Figure S7

Fig. S7. Imp and Syp binding sites display evolutionary linkage, related to Fig. 7.
(**A-B**) Comparison of PhastCons sequence conservation score of Imp and Syp binding sites from each iCLIP library versus the transcriptome average using (**A**) the 27-way *Drosophila* or (**B**) 124-way insect PhastCons track downloaded from the UCSC genome browser.
(**C-D**) Comparison of PhyloP sequence conservation score of Imp and Syp binding sites from each iCLIP library versus the brain transcriptome and the shuffled iCLIP binding sites per gene per transcript features using (**C**) the 27-way *Drosophila* or (**D**) 124-way insect PhyloP score matrices. For shuffled binding sites, the simulation was iterated 10 times per query. *$p<0.05$, ***$p<0.001$, ****$p<0.0001$.

Supplementary Data (separate files)

Supplementary Data S1: Summary of Imp and Syp iCLIP targets.

exp_string: iCLIP RNA-binding protein and developmental stage; gene_id: FlyBase gene ID (Ensembl release 99); gene_name: FlyBase gene name; gene_biotype; gene biological type; n_binding_site: number of significant RBP binding sites; binding_feature: iCLIP binding site transcript features; l2fc_xlsite: log2FoldChange of crosslinks compared to SMInput across the entire gene body; padj_xlsite: adjusted p-value of crosslinks compared to SMInput across the entire gene body; l2fc_bsmax: log2Foldchange of the most enriched binding site compared to SMInput; padj_bsmin: minimum adjusted p-value within binding sites compared to SMInput; feature_pct: percentage of iCLIP binding sites overlapping with each transcript feature; avg_iCLIP_tpm: average crosslink transcripts per million; human_homologs: high confidence human homologs (DIOPT score ≥ 8); mammalian_imp_target: conserved target with mammalian IMP1-3 in human pluripotent stem cells (*47*); mammalian_syp_target: conserved target with mammalian SYNCRIP or HNRNPR in rodent neurons (*48*, *49*).

Supplementary Data S2: GO enrichment analysis of Imp and Syp iCLIP targets.

GO_type: Gene Ontology (GO) class; exp_string: iCLIP RNA-binding protein and developmental stage; GO_id: GO accession number; Description: GO ontology term; GeneRatio: ratio of genes annotated with each GO term in the foreground set; BgRatio: ratio of genes annotated with each GO term in the brain transcriptome background set; p.adjust: adjusted p-value of the enrichment hypergeometric test; gene_names: annotated genes in the foreground set for each GO term; l2fc: log2FoldChange enrichment of each GO term.

Supplementary Data S3: Differential occupancy Imp and Syp targets.

gene_id: FlyBase gene ID; gene_name: FlyBase gene name; imp_target: L1_Imp or L2_Imp iCLIP target; syp_target: L2_Syp or L3_Syp iCLIP target; cluster_id: differential occupancy cluster grouping; cluster_description: differential occupancy cluster description; sypKO_l2fc: log2FoldChange of transcript expression comparing sypKO versus wildtype L3 brains; sypKO_padj: adjusted p-value of sypKO_log2fc.

Supplementary Data S4: Co-evolution analysis output between Imp and Syp binding sites.

gene_id: FlyBase gene ID; gene_name: FlyBase gene name; row_coverage: total nucleotide coverage of query binding sites; row_mean: mean mutual information score across total nucleotide coverage of query binding sites; row_median: median mutual information score across total nucleotide coverage of query binding sites; region_coverage: nucleotide coverage of Imp and Syp binding sites; region_mean: mean mutual information score across pairwise Imp and Syp binding sites; region_median: median mutual information score across pairwise Imp and Syp binding sites; tstatic: t-test statistics; pvalue: t-test p-value; comparison_mode: comparison direction from query to reference, either Imp-to-Syp or Syp-to-Imp; feature: comparison transcript feature, either UTR5 or UTR3.

Supplementary Data S5: smFISH probe sequences used in this study.

smFISH probe sequences were designed using the online Stellaris probe designer tool (https://www.biosearchtech.com/stellaris-designer) and ordered as unmodified oligonucleotides from IDT (see Methods).
